# Complete genome of streamlined marine actinobacterium *Pontimonas salivibrio* strain CL-TW6^T^ adapted to coastal planktonic lifestyle

**DOI:** 10.1186/s12864-018-5019-9

**Published:** 2018-08-22

**Authors:** Byung Cheol Cho, Stephen C. Hardies, Gwang Il Jang, Chung Yeon Hwang

**Affiliations:** 1 Microbial Oceanography Laboratory, School of Earth and Environmental Sciences and Research Institute of Oceanography, Seoul National University, Gwanak-gu, Seoul, Republic of Korea; 2 Department of Biochemistry, The University of Texas Health Science Center at San Antonio, San Antonio, TX USA; 3 Division of Life Sciences, Korea Polar Research Institute, Incheon, Republic of Korea

**Keywords:** *Pontimonas salivibrio*, Microbacteriaceae, Streamlined, Photoheterotroph, Coastal marine environment

## Abstract

**Background:**

*Pontimonas salivibrio* strain CL-TW6^T^ (=KCCM 90105 = JCM18206) was characterized as the type strain of a new genus within the Actinobacterial family Microbacteriaceae. It was isolated from a coastal marine environment in which members of Microbactericeae have not been previously characterized.

**Results:**

The genome of *P. salivibrio* CL-TW6^T^ was a single chromosome of 1,760,810 bp. Genomes of this small size are typically found in bacteria growing slowly in oligotrophic zones and said to be streamlined. Phylogenetic analysis showed it to represent a lineage originating in the Microbacteriaceae radiation occurring before the snowball Earth glaciations, and to have a closer relationship with some streamlined bacteria known through metagenomic data. Several genomic characteristics typical of streamlined bacteria are found: %G + C is lower than non-streamlined members of the phylum; there are a minimal number of rRNA and tRNA genes, fewer paralogs in most gene families, and only two sigma factors; there is a noticeable absence of some nonessential metabolic pathways, including polyketide synthesis and catabolism of some amino acids. There was no indication of any phage genes or plasmids, however, a system of active insertion elements was present. *P. salivibrio* appears to be unusual in having polyrhamnose-based cell wall oligosaccharides instead of mycolic acid or teichoic acid-based oligosaccharides. Oddly, it conducts sulfate assimilation apparently for sulfating cell wall components, but not for synthesizing amino acids. One gene family it has more of, rather than fewer of, are toxin/antitoxin systems, which are thought to down-regulate growth during nutrient deprivation or other stressful conditions.

**Conclusions:**

Because of the relatively small number of paralogs and its relationship to the heavily characterized *Mycobacterium tuberculosis*, we were able to heavily annotate the genome of *P. salivibrio* CL-TW6^T^. Its streamlined status and relationship to streamlined metagenomic constructs makes it an important reference genome for study of the streamlining concept. The final evolutionary trajectory of CL-TW6 ^T^ was to adapt to growth in a non-oligotrophic coastal zone. To understand that adaptive process, we give a thorough accounting of gene content, contrasting with both oligotrophic streamlined bacteria and large genome bacteria, and distinguishing between genes derived by vertical and horizontal descent.

**Electronic supplementary material:**

The online version of this article (10.1186/s12864-018-5019-9) contains supplementary material, which is available to authorized users.

## Background

Coastal environments at temperate regions represent varying conditions in many respects around the year: salinity can change due to river runoff, and oxygen concentration may shift to hypoxia in summer and from saturated concentration in bulk seawater to microoxic in biofilms and particles. In addition, pollutants can vary in compositions and quantities. To try to better understand the distribution and adaptations of bacteria in this environment, we have been culturing and characterizing bacteria from different niches within the coastal environment. One of these was the slow growing marine actinobacterium *Pontimonas salivibrio* CL-TW6^T^ (=KCCM90105 = JCM18206), which is the solitary representative of a new genus belonging to family Microbacteriaceae in the order Actinomycetales [1]. The family Microbacteriaceae is one of several large Actinobacterial groups including 46 genera and 289 species with validly published names [2]. Among them, only 4 type strains, including *P. salivibrio*, and only one species with a partially sequenced genome have been identified from coastal waters. *P*. *salivibrio* was isolated from a solar saltern pond having a slightly higher salinity than seawater near Jeung-Do (34° 59’ N, 126° 9′ E), Korea. However, it was found to grow in culture at normal marine salt concentration. Nutrient requirements, and other phenotypic characteristics have been determined [1, 3]. With only this one isolation site known, there is insufficient information to fully understand the habitat preference or ecology for members of *P. salivibrio*. However, we assume it was drawn into the saltern from some natural niche within the coastal environment.

Although Microbacteriaceae are rare in marine and particularly coastal environments, Actinobacteria in general are not. As of May 2018, there were about 240 marine Actinobacteria at some level of assembly in the JGI genome database, with *Salinispora* species being most dominant (63 genomes). The isolation sites range from coastal to deep sea and from seawater surface to sediment, and many are found in association with marine flora or fauna. Besides its taxonomic status, *P. salivibrio* was found to have other properties unexpected for Actinobacterial coastal marine isolates. It grows slowly, for example, by comparison to *Nocardioides* species in our collection [4, 5]. It utilizes a narrow selection of nutrients [1, 3]. Those properties suggest that *P. salivibrio* belongs to the paradigm of a streamlined specialist, as described further below. The sequencing study reported herein confirms that *P*. *salivibro* CL-TW6^T^ is streamlined.

Marine actinobacteria, as with other marine bacteria, can be thought of as generalists or specialists (for review, see [6, 7]). Generalists have large genomes, deal with larger numbers of secondary metabolites (metabolites outside of the most basic metabolic pathways), and tend to seek and exploit alternative nutrients with a strategy to consume and multiply rapidly. Specialists have small genomes which are said to be “streamlined”. The term “streamlined” is intended to not just mean a smaller genome, but to imply an evolutionary process during which nonessential genes are eliminated and metabolic processes are simplified. Streamlined bacteria deal with fewer secondary metabolites, are focused on fewer kinds of nutrient molecules, and tend to grow slowly. Streamlined marine bacteria tend to be free-living, meaning not associated with multicellular organisms, biofilm, or adhered to particles. Generalists are often targeted for characterization in order to find new drug candidates. Specialists are often invoked to support the theory that there are a large number of possibly uncultivable species of bacteria, particularly in oligotrophic (nutrient-poor) zones. An implication of the term “specialist” is that these streamlined bacteria may be more severely limited to specific niches. Among the best known cultured and characterized streamlined bacteria are *Pelagibacter ubique*, thought to represent a major portion of oceanic bacteria [8] and *Prochlorococcus* species, a major contributor to photosynthesis [9]. Among marine actinobacteria, streamlined representatives are mainly known through assembly of metagenomes [10]. There is a pressing need for more cultivatable bacteria with well characterized genomes to flesh out the theoretical understanding of this important class of bacteria.

The length of the sequenced *P. salivibrio* CL-TW6^T^ genome reported here is 1,760,810 bp. This puts it in the range of streamlined specialists, consistent with the slow growth and narrow nutrient status. However, it lives in a zone not usually considered to be oligotrophic. For these reasons, we chose to do an extensive bioinformatics study on this genome. We present a detailed analysis of *P. salivibrio* gene content in the context of exploring how the streamlining process may reflect adaptations that specialize the species to niches found in coastal waters. This study seeks to look back in time rather than comparing broadly across all related species and genera. Hence we choose representative completely sequenced genomes of progressively greater distance to reconstruct descent of the lineage, finding that streamlining of the lineage mainly occurred in the 0.5–1.5 Gya time zone. This is a time during which substantial global upheaval affected marine environments [11–13]. We used a variation of BlastP-based core/auxiliary gene analysis to estimate that up to one half of *P. salivibrio* genes may have been acquired or replaced by horizontal transfer during that time.

## Results and discussion

### Additional phenotypic characterization

Additional phenotypic characterization was conducted (Additional file 1: Table S1), which were extensions of and generally consistent with the extensive characterization given by Jang et al. [1].

### General properties of the genome

The genome of *P. salivibrio* was sequenced and found to be a circular sequence of 1,760,810 bp [GenBank accession CP026923]. This is in the size range typically called streamlined, and observed in oligotrophic and/or planktonic bacteria (Table 1). No second bacterial chromosome or small plasmids were detected. There was a second sequence of 31,069 bp detected at about 2% of the stoichiometry of the *P. salivibrio* genome. That smaller genome, by sequence analysis, was clearly a lytic phage genome with a circular map and no indication of integration into the bacterial chromosome. For confirmatory sequencing, a second preparation of *P. salivibrio* DNA was made. In that second data set, the phage sequence completely disappeared, hence this is a phage that was poorly adapted to grow on this strain and is no longer available for further study. The phage sequence was deposited in GenBank [MG835450] by the name phiPsal1, and will not be further discussed here.

**Table 1:** Representative genomes used in comparative study

Strain	Taxonomy^a^	Accession number	Genome size (Mb)	Ecological niche	Reference
*Pontimonas* *salivibrio* CL-TW6^T^	Micrococcales Microbacteriaceae	CP026923	1.76^b^	coastal waters planktonic	This study [1]
acMicro-4^c^	Micrococcales family unspecified	JNSD01000000	1.21^b^	freshwater planktonic	[15]
*Microcella* *alkaliphila* JAM AC0309	Micrococcales Microbacteriaceae	AP017315	2.70	deep sea sediment^d^	[91]
*Yonghaparkia* sp. Root332	Micrococcales Microbacteriaceae	NZ_LMCX00000000	> 1.6	Plant root/ground water	[92]
*Clavibacter* *michiganensis* *nebraskensis* NCPPB 2581	Micrococcales Microbacteriaceae	NC_020891	3.06	plant pathogen	unpublished See [93]
*Leifsonia xyli cynodontis* *DSM 46306*	Micrococcales Microbacteriaceae	NC_022438	2.68	plant pathogen	[94]
*Microbacterium* *testaceum* StLB037	Micrococcineae Microbacteriaceae	NC_015125	3.98	endophytic	[95]
*Rathayibacter* *toxicus* 70137	Micrococcales Microbacteriaceae	NZ_CP010848	2.33	plant pathogen	unpublished See [96]
*Rhodoluna lacicola* MWH-Ta8	Micrococcales Microbacteriaceae	CP007490	1.43^b^	freshwater planktonic	[97]
*Mycobacterium* *tuberculosis* H37Rv	Corynebacterineae Mycobacteriaceae	NC_000962	4.41	human pathogen	[14]
*Corynebacterium* *glutamicum* WM001	Corynebacteriales Corynebacteriaceae	NZ_CP022394	3.31	soil, water	unpublished See [98]
*Streptomyces* *cattleya* DSM 46488	Actinomycetales Streptomycineae	NC_017586 NC_017585	6.28 1.81	soil, decaying vegetation	unpublished See [99]
MedAcidi-G1^b^	Acidimicrobiales	JUEM00000000	1.85-2	Mediterranean sea chlorophyll max.	[10]
*Prochlorococcus marinus MD4*	Cyanobacteria	NC_005072	1.66^b^	ocean, oligotrophic zone	[100]
*Pelagibacter ubique HTCC1062*	Alpha Proteobacteria	NC_007205	1.31^b^	ocean, oligotrophic zone	[8]

^a^Arranged in order of increasing phylogenetic distance

^b^Considered streamlined

^c^For metagnomic assemblies, only the assembly with the most similar genes to *P. salivibrio* was considered

^d^Although other members of *Microcella alkaliphila* have been found in fresh water [101]

Between the two chromosomal sequence determinations there was a missing copy of an insertion sequence (IS) in the second that was present in the first. That IS was present at 85% allele frequency in the first sample based on the number of reads showing an inserted site versus an uninserted site. That IS was included in the annotated sequence (ISPsa1). Other insertions of the same IS sequence present at lower allele frequency at other sites were also observed in the read data. Those were not included in the annotated sequence, but indicate that the IS copy number was actively changing during the culturing of the cells. We believe that the heterogeneity of IS elements was due to ongoing transposition rather than excision, because there were not substantial direct target site repeats suitable to support precise excision.

### Feature annotation

A first draft for locations of open reading frames (ORFs) and genes encoding RNAs was obtained from two annotation pipelines and then refined by a battery of more exhaustive analytical methods (see Methods). Analyses conducted included, in addition to the usual NCBI model set, hhpred searches of all hidden Markov model (HMM) protein family libraries we could access, including those derived from PDB, COGS, Pfam, CDD, and TIGR. To make BlastP more powerful in supporting feature annotation, the NCBI library was remade of all proteins from all completely sequenced bacterial genomes so that exact genome, coordinates, and any documenting notes were retained in the definition lines. This allowed synteny to be apparent from blast results without further processing, which was invaluable for certain genes that move in pairs, for example toxin/antitoxin systems and two component sensor systems. Retention of documentation notes was particularly valuable for matches to *Mycobacterium tuberculosis* H37Rv [14], the only GenBank entry within the phylum containing copious PubMed references for genes that have been biologically characterized. Those references often solved the common problem of sorting out which gene among a series of paralogs carries out a specific function. In our annotation, we marked a gene as orthologous to an H37Rv gene (and presumably having the same functional specialization) if the *P. salivibrio* gene found the H37Rv gene as the strongest BlastP match of any H37Rv gene, and also the H37Rv gene used as query found the *P. salivibrio* gene as its strongest match. This quick estimate of orthologs might be expected to be error prone in cases where there are many homologs of roughly equal similarity, so we avoided using it in those cases. With the use of orthologs to characterized H37Rv genes, and the relatively low number of paralogs in the streamlined *P. salivibrio* genome, nearly all *P. salivibrio* genes for most of the basic metabolic pathways were uniquely assigned (Additional file 2: Table S2). A summary of key pathways is shown (Additional file 3: Figure S1). Note: as of this writing the NCBI RefSeq version of our sequence does not contain our annotation. The annotation corresponding to our work product is only found in CP026923.

In the *P. salivibrio* genome, we annotated a total of 1698 ORFs. These include 43 ORFs exhibiting no BlastP matches of any kind. These could be either coding or noncoding sequence. The terms “hypothetical” and “putative” were used sparingly to indicate especially speculative assignments of function. The overall sequence was 58.3% G + C, consistent with being a member of the high %G + C Actinobacteria phylum. Also annotated were 46 tRNA genes, three rRNA genes, genes for signal recognition particle, tmRNA, the gene for the RNA component of RNAseP, and seven miscellaneous RNA structures such as riboswitches.

### Biogeographical and phylogenetic relationships

**Fig. 1:**
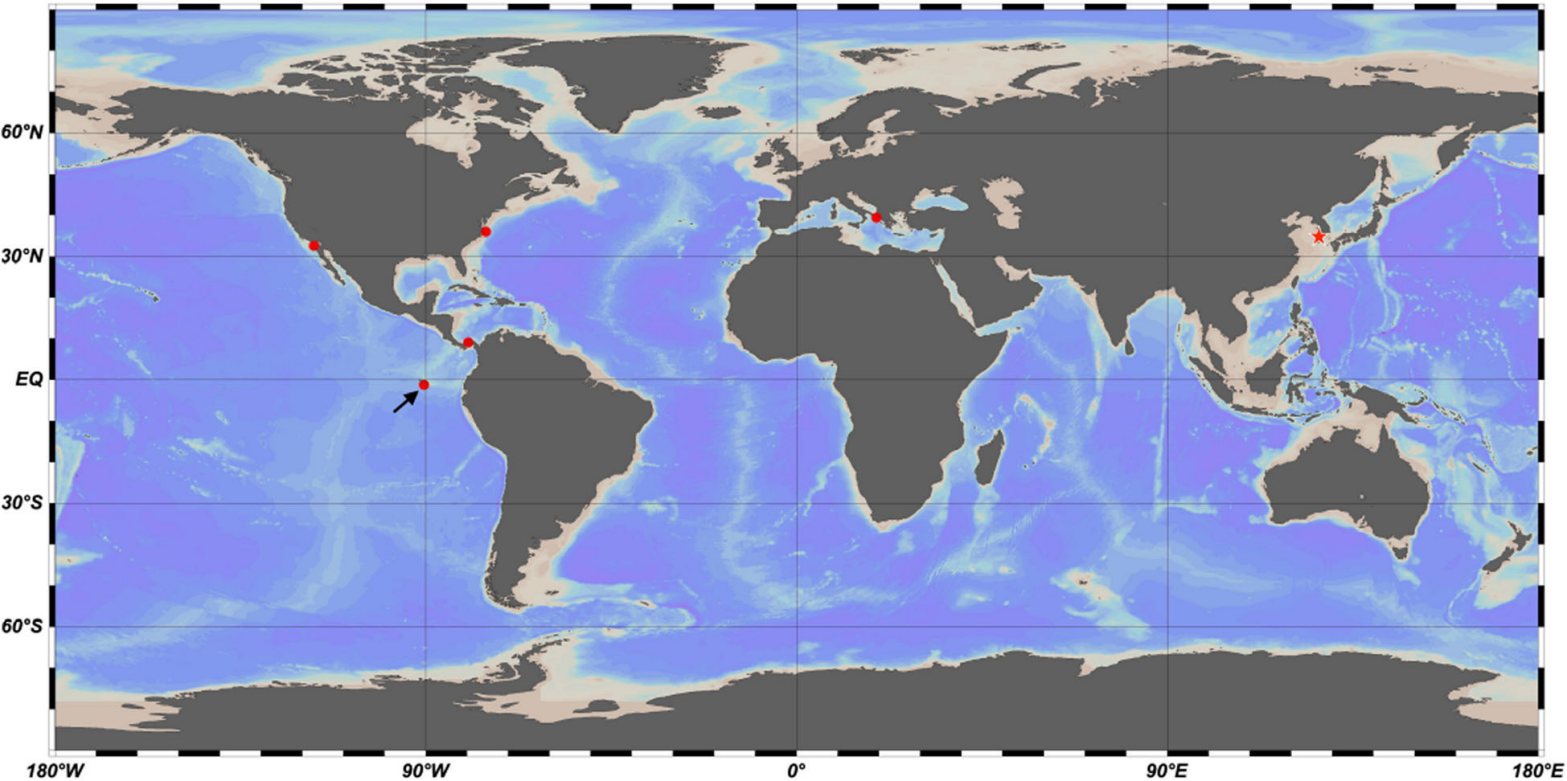
Geographic localization of metagenomic sequences with high similarity to *P. salivibrio* 16S rRNA. The point west of South America indicated by an arrow was for a metagenomic sample taken from a hypersaline lagoon (63.4 psu) at 0.2 m depth in the Galapagos Islands. The DnaE metagenomic sequence shown in Fig. 2 also came from this site. The star is the site of isolation of *P. salivibrio* CL-TW6^T^. The figure was produced by the Ocean Data View Tool (see Methods)

The 16S rRNA sequence has been used to place *P. salivibrio* in the Actinobacterial family Microbacteriaceae [1]. The most similar rRNA sequences in the MG-RAST and representative global ocean metagenome databases were mapped as to biogeographical locations (Fig. 1). These indicated a distribution globally over coastal locations of genomes related to *P. salivibrio*. The sampling is insufficient to determine if relatives are present on all coastlines or just some coastlines. The degree of relationship of these to *P. salivibrio* is clarified below as ranging from other species within *Pontimonas* to members of a closely related genus. Equally similar rRNA matches were not found in mid oceanic locations, or in non-marine locations.

An updated 16S rRNA tree (Additional file 4: Figure S2) shows some clustering of *P. salivibrio* with other Microbacteriaceae genera *Yonghaparkia*, *Microcella*, and *Rhodoluna*, although without strong bootstrap values. Analysis by average amino acid identity (AAI) confirms that *P. salivibrio* is at a level of divergence consistent with its assignment as a distinct genus from these or any other completely sequence member of Microbacteriaceae (Additional file 5: Table S3).

A collection of genomes was assembled to support all of the more detailed comparative study to *P. salivibrio* to follow. Table 1 gives accessions, references and other defining information for these genomes. Additional file 6: Table S4 gives parameters often discussed with respect to streamlining, and also the frequency with which each genome was found as a top BlastP match to a *P. salivibrio* encoded protein. The genomes were chosen to represent increasing phylogenetic distance to enable reconstruction of the evolution of the lineage, and to include a mixture of streamlined and non-streamlined genomes for comparison. Included were: 1) the genomes with the highest numbers of BlastP hits observed in general BlastP searches, 2) other selected Microbacteriaceae, 3) the acMicro-4 metagenome assembly which has been described as an example of streamlining, but not well classified taxonomically [15], 4) the heavily annotated *Mycobacterium tuberculosis* and a taxonomically-related free-living Corynebacterium, 5) a representative large genome bacterium from *Streptomyces*, 6) an additional metagenomic assembly, MedAcidi-G1, from Actinobacteria with a published analysis indicating streamlining, and 7) the classic streamlined genomes from *Prochlorococcus* and *Pelagibacter*. The average of the representative Microbacteriaceae is already partially streamlined. By comparison the average genome size of all completely sequenced Microbacteriaceae is 3.2 Mb, and of all Actinobateria is 4.0 Mb (not shown). Additional file 6: Table S4 indicates that within the actinobacterial representative genomes, %G + C is correlated with streamlining with *P. salivibrio* and other streamlined Actinobacteria on the low %G + C side of this generally high %G + C phylum. We examined interORF spacing (data not shown), but did not observe a significant trend correlated with streamlining. There was, however, a significant streamlining effect on the numbers of tRNA and rRNA genes.

**Fig. 2:**
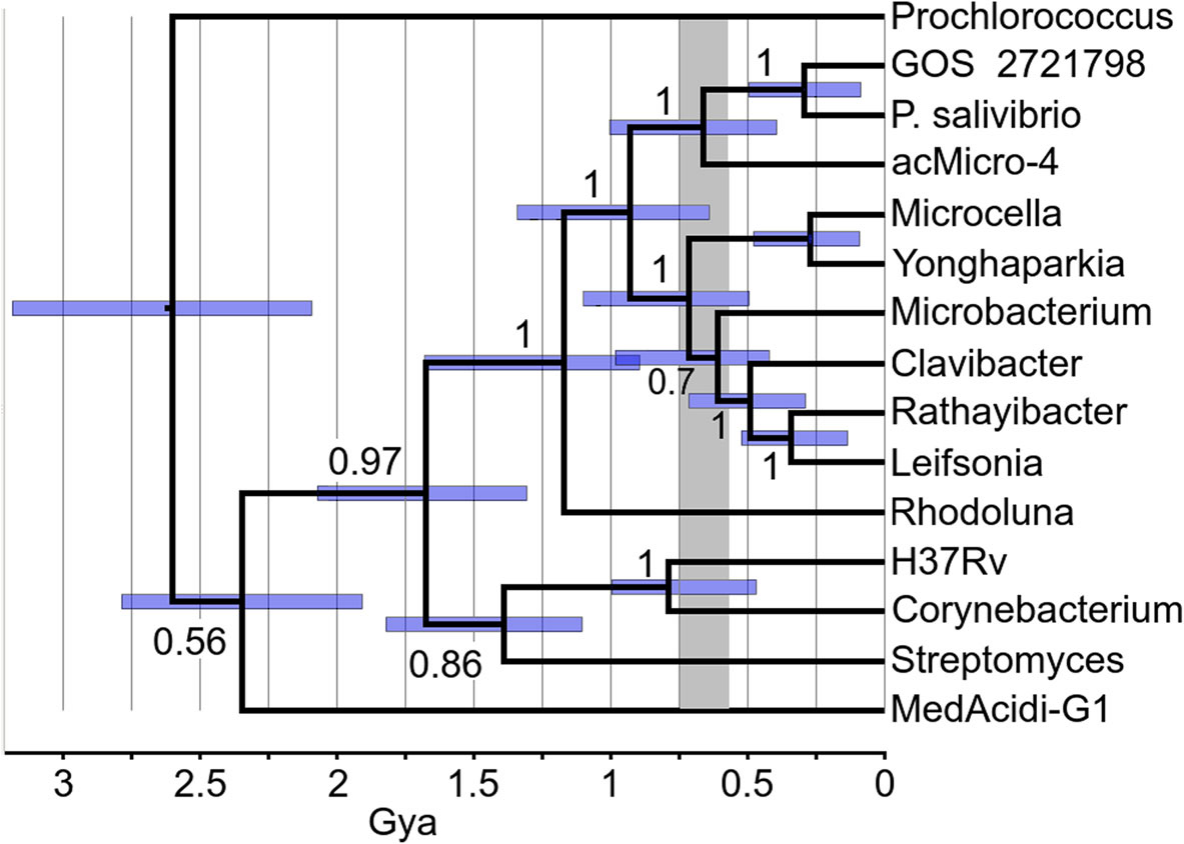
Maximum likelihood tree of the DnaE gene. The timescale was chosen to most closely align with H37Rv/*Corynebacterium*, 0.95 Gya; H37Rv/*Streptomyces*, 1.4 Gya [102]. Posterior probabilities of the nodal splits are shown numerically. The blue bars represent the 95% probability interval for node heights. The gray bar represents the timing of snowball Earth [11]. GOS 2721798 is a metagenomic sequence marked by the arrow on Fig. 1

We wanted to know if the rRNA results or the more frequent top BlastP matching of other genera like *Yonghaparkia, Microcella,* or *Rhodoluna* and/or of the acMicro-4 metagenome assemblage represented closer ancestry. If a closer common ancestor than that of all Microbacteriacea can be found even at the subfamily level, evolutionary reconstruction could then comment on the progress of streamlining at that point. Using tree-making methods, a closer relationship with the acMicro-4 assembly was confirmed, but a closer relationship with *Yonghaparkia*, *Microcella* or *Rhodoluna* was rejected. In our strongest attempt to try to force a relationship with *Yonghaparkia*, *Microcella* or *Rhodoluna*, twenty six large proteins having that BlastP matching preference were examined by tree construction with PAUP (not shown). A subset of nine genes that did not have other congruency issues was subjected to a phylogenomic tree by the maximum likelihood method (Additional file 7: Figure S3). None of that supported a closer relationship with *Yonghaparkia*, *Microcella*, or *Rhodoluna* and the discrepancy between blast ranking and the trees was not further pursued. All of the tree work supported a closer relationship with acMicro-4. A representative single gene tree (DnaE, C3B54_11990) is shown in Fig. 2. AAI between *P. salivibrio* and acMicro-4 is at the top end of the range for inclusion within the same genus (Additional file 5: Table S3). However, for consistency with the classification of the other Microbacteriaceae it might be considered as separate closely related genus. The size of the acMicro-4 genome has been estimated at 1.21 Mb suggesting that the common ancestor to acMicro-4 and *P. salivibrio* was already essentially finished with streamlining.

The tree was made with a time scale to place streamlining into context with events affecting the earth over the same time. Figure 2 shows that the streamlined ancestor to acMicro-4 and *P. salivibrio* was already in place either before or associated with a global upheaval called “snowball Earth” [11–13]. Prior to this time, plants or animals of the kind found in association with some of the Microbacteriaceae had not yet evolved. The food chain in the photic zone was based on cyanobacteria, whether in fresh or salt water. During this time, there were several lengthy bouts of global glaciation. It is controversial how photosynthetic and phototrophic microbes survived and conserved those features during the lengthy glaciations. Various proposals invoke water exposed in equatorial refuges, volcanic hot spots, photosynthesis under thin ice or slush, or by bacteria mixed into or on the surface of ice. In any case, the biogeographical niches currently occupied will not have become available until the ice cleared. The metagenomic rRNA sequences of Fig. 1 cannot be placed on a tree together with acMicro-4, because there is no intact 16S rRNA sequence in the acMicro-4 assembly. Instead, a metagenomic DnaE sequence was found (GOS2721798) colocated with one of the geolocated rRNA samples in Fig. 1 and added to the tree in Fig. 2. The next most similar class of metagenomic DnaE sequences joined at the same time as acMicro-4 (not shown). Hence GOS2721798 is clearly representative of another *Pontimonas* species, but the other metagenomic finds could ambiguously be considered either *Pontimonas* or members of a related genus. This clarifies when the biogeographical distribution shown in Fig. 1 was created in time. It reflects the radiation of the *P. salivibrio* lineage as it emerged from snowball Earth. During this period, coastal waters were thought to have been especially enriched in nutrients as a result of glacial outflow [13]. Therefore, the major evolutionary process in the last 0.5 Gya for *P. salivibrio* was expansion from an austere snowball Earth environment into a richer coastal water environment. The data cannot exclude that streamlining may have stopped or even reversed during those last 0.5 Gyr.

Core gene analysis was conducted dividing the genes into the core (670) found by BlastP in all of the Microbacteriaceae and auxiliary genes found in some but not others (not shown). This gives a rough separation between genes mainly transmitted vertically (the core) and genes engaged in some mixture of gene loss and horizontal exchange. We refined this in two ways (see details in Methods). First, we retained for each gene a parameter indicating the frequency of BlastP hits in the Microbacteriaceae library. An auxiliary gene found in all but one genome, for example, would be expected to be most likely to have descended by vertical descent in *P. salivibrio* and either lost or replaced by horizontal transfer in one of the other Microbacteriaceae*.* Conversely, a gene found in no other Microbacteriaceae almost certainly arrived by horizontal transfer, and those found in an intermediate number have a mixed likelihood of vertical descent versus horizontal transfer. We called this parameter the “vertical index” and use it to identify genes in pathways and other ensembles that may have been modified in response to adaptive pressure during the journal of the lineage into its final niche. Secondly, we were aware of incongruencies among core gene single gene trees from the above tree-making exercise, indicating that core genes are not purely descended by vertical transmission. This happens because core genes are often essential and cannot be lost. So, when a horizontal transfer provided an alternative homolog from a far-away taxon, it is a replacement, and is still found by BlastP. To expose that sort of horizontal transfer, we altered the counting of BlastP matches to only those matching to in the top 10 matches to Microbacteriaceae. This was done in a library of only complete genomes, covering the whole of the bacterial domain and containing 10 Microbacteriaceae genomes. This amounts to a congruency check on any gene horizontally transferred from a far-away taxon since the ~ 1.5 Gya point on the tree. The core with this congruency check dropped from 670 to 360 genes. The results are reported as a vertical index of 0 to 10 (Additional files 2 and 8: Tables S2 and S5). This table is used extensively below to flag genes in pathways that appear to have been altered during the time of streamlining or the subsequent adaptation to *P. salivibrio*’s current environmental niche.

The relationship of low vertical index to the concept of genomic islands was considered. A plot by genomic location (Additional file 9: Figure S4) shows a number of clusters of genes with low vertical index, the largest of approximately over 150 consecutive genes (C3B54_111226–111383). The Islandviewer tool [16] scores that region as unusual in dinucleotide composition (not shown) and it contains several IS elements. There may be one or more block insertions of the kind called “genomic islands” in that region, and in smaller regions of low vertical index. There were no accumulations in these regions of prospective pathogenicity genes, or prophage genes. In fact, we could not find a single gene in this genome that we could confidently call “phage-derived”. The prospective genomic island regions were rich in transporters, toxin/antitoxin systems, enzymes not strongly associated with well characterized pathways, and also contained a restriction system. The nucleotide signatures characteristic of genomic islands is expected to fade within a few Myr given the synonymous mutation rate for bacteria [17]. Hence these regions can reflect only the most recent adaptive struggle for niche fulfillment.

Within the library of completely sequenced bacterial genomes, 488 had none of their top 10 BlastP matches to any member of Microbacteriaceae, and another 421 had only 1 to 3 of their top 10 matches to Microbacteriaceae. Examination of the BlastP rankings of the Microbacteriaceae in the low vertical index classes reveals indications of horizontal transfer in those classes. For example, a *P. salivibrio* gene may match only other Microbacteriaceae genomes but at a level of similarity equilivant to matches to far-away taxon, suggesting independent acquisitions by horizontal transfer. We estimate that the total fraction of genes acquired by horizontal transfer was between 1/4 and 1/2 of the genome. Most of these are not in prospective genomic islands, but interspersed with the vertically descended genes (Additional file 9: Figure S4). We believe this reflects the history of genes acquired and retained over the full 1.5 Gyr history since the common ancestor of Microbacteriaceae.

### Genes related to photoheterotrophy

Among the annotated genes, one that may be of particular relevance to the marine character of *P. salivibrio* was a bacteriorhodopsin (C3B54_11643). This protein, through pumping protons under the power of light exposure, potentially augments ATP production in an oligotrophic environment (photoheterotrophy). By analysis at the MicRhoDE server, C3B54_11643 was a member of the green light absorbing Exiguobacterium subcluster. The specific residue motif thought to tune it to green light [18] is present. It did not cluster with other bacteriorhodopsins found in Actinobacteria, sometimes called actinorhodopsins. The relevance of green light sensing is that green light only penetrates the first 10 to 20 m of water; deeper bacteria must use a blue light-absorbing rhodopsin. This is consistent with location in shallow coastal waters. The analysis below indicates that the *P. salivibrio* bacteriorhodopsin was conserved over the last 1 Gya, suggesting that the niche has consistently been a shallow water niche over that time.

The namesake, Exigoubacterial rhodopsin, has been characterized as a poor proton pumper and instead as a light sensor linked by signal transduction to a locomotion system [19]. However, *P. salivibrio* has no locomotion system, and we were unable to find a homolog to the Exiguobacterial transduction system in *P. salivibrio*. Hence, we believe that the function of the *P. salivibrio* rhodopsin is photoheterotrophy. Of the representative genomes, an ortholog was found in acMicro-4 and *Yonghaparkia*, and a different bacteriorhodpsin is found in *Microcella*, *Rhodoluna*, MedAcidi G1, and *Pelagibacter*. All of the representative genomes from an aqueous environment had either photoheterotrophy or photosynthesis. *Microcella* and *Yonghaparkia* also had photoheterotrophy. The *Microcella* isolate was from deep sea sediment, although the species has also been reported from fresh water. The *Yonghaparkia* isolate was from plant roots, which would be in interaction with ground water. We suggest that the presence of the bacteriorhodopsin acts as a marker for dispersal of the species from light exposed water within that period of evolutionary time over which an unused gene would be lost.

The orthology of the *Yonghaparkia* and acMicro-4 rhodopsins was confirmed by their occupancy of syntenic locations relative to *P. salivibrio* (data not shown). Their relative divergence values were consistent with the topology of Fig. 2. This version of the rhodopsin was therefore probably the ancestral version existing prior to snowball Earth, consistent with a role of photoheterotrophy in the partial streamlining already existing in that ancestor. During snowball Earth, some lineages maintained this gene implying continuous access to an adequate light source to conduct photoheterotrophy. Other lineages lost it, and others exchanged it either during or after snowball Earth, as might be expected for the chaotic environmental conditions that these lineages traversed. *P. salivibrio* has four photosensitive lyases. One of these (C3B54_11283) exhibits vertical descent with sufficient synteny in the group *Yonghaparkia*, acMicro-4, and *P. salivibrio* to again indicate continuous light exposure during the evolution of that group. The UvrABC locus (C3B54_111064–66) showed more extensive vertical descent, although that enzyme may repair defects other than UV-induced ones.

The genes for the synthesis of retinal and other isoprenoids have been assigned (Additional file 2: Table S2, isoprenoid). The overall pathway shows extensive conservation with mainly vertical descent from Microbacteriaceae ancestors. However, the last three genes, which determine the production of alpha and beta carotene and retinal were derived since the separation from acMicro-4 by horizontal acquisition of an operon from outside of the family. The retinal would have been required over the whole time when rhodopsin was present, and pigmentation by carotenes was essential for UV protection [20], even more so prior to snowball Earth when the ozone layer was not yet developed. So this horizontal transfer was a replacement of function, not an acquisition of new function. The variation in optimal output of the pathway might have been a force to drive the horizontal replacement.

### Genes related to planktonic vs. biofilm life style

Streamlining, photoheterotrophy, lack of motility, and planktonic life-style are thought to be correlated, at least in oligotrophic zones [10]. Correspondingly, *P. salivibrio* lacks any genes for production of flagella. However, it has two fimbriae structural gene operons as well as genes involved in fimbriae production in its genome (Additional file 2: Table S2, planktonic). These genes appear to have been inherited from Microbacteriaceae ancestors. Hence, whereas *P. salivibrio* has not been observed to form biofilm itself, there may be circumstances where it binds particles [21, 22] or joins biofilm generated by other bacteria. No genes for generation of a quorum signal were identified. However, an autoinducer-2 (AI-2) quorum sensing operon was present (Additional file 2: Table S2, planktonic), which suggests that it can detect and respond to the presence of biofilm. The quorum sensing system had low vertical index indicating a recent acquisition. The *P. salivibrio* genome did not have a diguanylate cyclase gene, which is involved in regulating the transition from planktonic to biofilm forms in bacteria that make that transition [23]. Hence *P. salivibrio* probably does not alter expression of large numbers of genes and enter a differential biofilm state like many bacteria do. Another possibility is that *P. salivibrio* might take up AI-2 not to respond to it but rather to destroy it, disrupting biofilm formation by other bacteria [24].

### Cellular regulators - sigma factors

*P. salivibrio* encodes only two sigma factors (Additional file 2: Table S2, sigma). Both are of the sigma-70 family. In relation to the well characterized collection of sigma factors in H73Rv [25], one is orthologous to the primary housekeeping sigma factor SigA (Rv2703), and the other is orthologous to the extracellular function (ECF) sigma factor SigH (Rv3223c). Although ECF factors are named because of the tendency to include extracellular functions in their regulons, most of the ~ 180 function regulated by H37Rv SigH are intracellular. More meaningfully, the SigH sigma factor is regulated by an antisigma factor and signaling system responsive to stress, and which appears to be conserved in *P. salivibrio*. In H37Rv, SigH responds to heat shock, and oxidative stress. The number of paralogs of sigma factors varies widely in the set of representative genomes from a minimum of 2 to a maximum of 51, with the ECF set being the largest and most varied number (Additional file 10: Table S6, sigma). Streamlined genomes generally encode fewer (2–5) (see also [10]). The Microbacteriaceae ancestors to *P. salivibrio* appear to have had an average of 9. How and why a genome loses so many layers of regulation during streamlining is an unexplored theoretical question, but it seems that the basal state is one housekeeping sigma factor and one generic stress response sigma factor.

### Cellular regulators - two component systems

A widely used class of membrane signal kinases in bacteria are the two component systems consisting of a sensor histidine kinase and a cytoplasmic response regulator [26]. There are generally fewer two component systems in streamlined bacteria (Additional file 10: Table S6, two component), suggesting that streamlined bacteria have a lessor capacity to respond to environmental signals. The number in *P. salivibrio* (11) are on the low side for non-streamlined Microbacteriacea (13–36) but on the high side for other streamlined members (4–11). This numerology of being low compared to Microbacteriaceae but high compared to streamlined genomes is typical of other paralogous families (see below). Of the 11, five two component systems exhibit relatively vertical descent and can be identified with characterized two component sensors in *Mycobacterium tuberculosis* (Additional file 2: Table S2, two component). These have been proposed to respond to Pi limitation, iron limitation, to sense the state of cell wall synthesis, to regulate lipid metabolism, or to respond to osmotic stress [27–29]. The others appear to have arisen by horizontal transfer. In many cases the only evidence that the kinase and sensor are functionally paired is that the regulator is encoded upstream of the kinase. We confirmed the conservation of that arrangement by mapping the most closely related homologs in other genomes. There were a few solo kinases and regulators. We assigned one functional pair (C3B54_111603, C3B54_111071) among these unlinked genes because their orthologs in H37Rv have been biochemically demonstrated to interact. That particular pair exhibits the most consistent vertical descent of the family. Hence, the *P. salivibrio* two component systems show a mixture of conserved functions and functions adopted by horizontal transfer during adaptation to the current environment.

### Cellular regulators - toxin/antitoxin systems

Another major class of regulators are the toxin/antitoxin systems. These were originally discovered as a pair of genes where one carried by a plasmid was necessary to prevent killing by the other on the bacterial chromosome. However, a more modern interpretation is that these are used to sense a stress condition and place the bacteria in a persistent non-growing state to enhance survival of the stress. *Mycobacterium tuberculosis* is known for especially high numbers of toxin/antitoxin systems [30]. This family of regulators does not follow the trend for streamlined bacteria in *P. salivibrio*. It has the second highest number of toxin/antitoxin systems (24) in the set of representative genomes, lower only than *Mycobacterium tuberculosis* (Additional file 10: Table S6, toxin/antitoxin). In *Mycobacterium tuberculosis*, the high number is interpreted as indicating a greater diversity of challenges that the strain is required to negotiate [30]. In *P. salivibrio*, all but one of the systems was acquired by horizontal exchange (Additional file 2: Table S2, toxin/antitoxin). So, the toxin/antitoxin systems have been intensively revised during adaptation to the current environment. *P. salivibrio* has two Maz EF systems, which are thought to respond to nutrition deficiency by stringent control [30]. Other components of the stringent control system are tabulated (Additional file 2: Table S2, toxin/antitoxin). The other representative streamlined genomes, in contrast, have very few toxin/antitoxin systems (0–2). Apparently *P. salivibrio* has some special reliance on toxin/antitoxin systems for regulating its slow growth life style other than what occurs in oligotrophic bacteria thus far characterized.

### Maintenance of reducing environment

As is generally the case in Actinobateria, glutathione is not synthesized in *P. salivibrio* (Additional file 10: Table S6, redox). The redox agent mycothiol, present in many Actinobacteria [31], is not sythesized in *P. salivibrio*. As judged by mycothiol synthase as a marker enzyme, mycothiol is used sparingly in Microbacteriaceae and never in streamlined genomes (Additional file 10: Table S6, redox). Instead *P. salivibrio*, and many other Microbacteriaceae, keep cysteines of cytoplasmic proteins reduced using CoA disulfide reductase [32].

*P. salivibrio* has a thioredoxin system which may also directly reduce disulfide bonds in cytoplasmic proteins, but also provides reducing power to a number of cellular reductases, including oxidative stress response enzymes [33]. Of the nine proteins with thioredoxin similarity, C3B54_11146 is orthologous to the major thioredoxin of H37Rv, and three (C3B54_116, C3B54_111696, C3B54_11616) perform functions other than providing reducing power as detailed (Additional file 2: Table S2, redox). The other five thioredoxin paralogs without established function are candidates for auxiliary or stress-induced thioredoxins with moderate to high tendency for involvement in horizontal exchange. The total numbers of thioredoxin-like domains is similar to the other representative genomes, and there appears to be little evidence for economization of thioredoxin systems in streamlined strains (Additional file 10: Table S6, redox).

### Response to oxidative stress

*P. salivibrio* failed to grow in culture in medium supplemented with 1 mM H_2_O_2_ (Additional file 1: Table S1). Other marine bacteria have been observed to grow with H_2_O_2_ as high as 25 mM [34]. It is not clear how the sensitivity to an external H_2_O_2_ corresponds to the physiological oxidative stress challenge, which is likely to be generation of H_2_O_2_ produced by interaction of O_2_ with the electron transport chain and with free iron in the cell. The internal level of H_2_O_2_ in *E. coli* from this source is thought to be around 20 nM, with raised levels to the micromolar range caused by loss of defensive enzymes leading to measurable genetic damage [35]. We looked for any omission from the standard toolkit for handling oxidative stress in *P. salivibrio*. The best characterized member of Actinobacteria for handling oxidative stress is *Mycobacteria tuberculosis* [36]. The Mycobacterial regulatory proteins and major responders, catalase and superoxide dismutatase, appear to be conserved in *P. salivibrio*. *P. salivibrio* has a similar number of peroxidases to those annotated in H37Rv, although they are a different selection from the diverse families available. One possible deficiency is in the dps protein, which is a scavenger of free iron and which is induced by oxidative stress. The dps version in *P. salivibrio* is foreshortened, and may be a pseudogene. There are five DNA repair glycosylases that might repair DNA after oxidative damage in H37Rv, and *P. salivibrio* has a similar number.

### Hypoxia

Coastal waters are prone to develop hypoxia [37]. The major identifiable resource in the *P. salivibrio* genome for dealing with hypoxia is a cytochrome bd terminal oxidase (C3B54_11787/C3B54_11788) for the respiratory chain. The bd complex is a separate terminal branch from the bcc-aa3 complex (C3B54_111003/C3B54_111004/C3B54_111005/C3B54_11998/C3B54_11999/C3B54_111000/C3B54_111006) which reduces O_2_ and pumps protons under normoxic conditions. The bd complex is optimized for low O_2_ with a higher O_2_ affinity. The electron transfer branch leading to bd also pumps fewer protons which may enable respiration to keep running with the lower free energy available to drive it at low O_2_. Both *P. salivibrio* bcc-aa3 and bd complexes have orthologs in H37Rv. High affinity terminal oxidases, like bd, are widely distributed in nature, but are not found in open ocean planktonic bacteria, a fact attributed to streamlining [38]. The other streamlined members of the representative genomes have lost the bd complex (Additional file 10: Table S6, redox). Its retention in *P. salivibrio* is interesting both because it makes sense in its environment to keep this function, but because it apparently vertically descended through streamlined ancestors. There is also a bacterial hemoglobin (C3B54_11733) thought to aid respiration at low O_2_.

### Heat and cold stress

Growth of *P*. *salivibrio* occurs at 15–35 °C (optimum of 30 °C) [1]. In coastal waters, water temperature falls to 7 °C in winter, but does not go higher than 35 °C in summer. From this profile, it is unclear how important the heat shock response would be. None-the-less, *P. salivibrio* conserves two heat shock regulons (Additional file 2: Table S2, heat/cold). The Hsp70 operon including the HspR heat shock repressor, and chaperones DnaK, GrpE, DnaJ, and ClpB is conserved syntenically with H37Rv. The Hsp65 regulon, although not organized as a contiguous operon, is represented by heat shock repressor HrcA, DnaJ2, GroEL and GroES. Proteases involved in the heat shock response include Lon (cytoplasmic), HtpX (membrane), and DegP (periplasmic). These destroy denatured proteins generated during heat shock and are induced themselves by heat shock in at least some bacterial species. However, the S4 ribosome subunit recycling factor known as hsp15 and the inclusion body binding proteins represented by H37Rv hspX are not represented in *P. salivibrio*. Examination of number of paralogs (Additional file 10: Table S6, heat/cold) reveals that *P. salivibrio* has on average a few genes less devoted to heat stress response than the other representative genomes.

*P. salivibrio* encodes orthologs of two H37Rv cold shock RNA-binding chaperones, and an ortholog of the *B. subtilus* cold shock ATP-dependent RNA helicase, CshA [39]. These play a role in removing unhelpful RNA secondary structure at low temperature. There are two additional ATP-dependent RNA helicases which may play similar roles. The gene for ribosome-binding factor A, which aids ribosome assembly at low temperature [40] is present. NusA, involved in multiple stress responses and a cold shock protein [41], are present. So are polynucleotide phosphorylase [42] and fatty acid desaturase [43]. In addition to fatty acid desaturation, content of branched chain fatty acids (see below) may be an important factor governing membrane fluidity at low temperature.

### Osmoregulation

Bacteria in coastal waters experience variable osmotic stress due to on proximity to and variation in inflow of fresh water. *P. salivibrio* survives in salt concentrations between 1 and 5% [1], sufficient to explain its presence in a saltern, but not tolerant in the range reached by some extreme halophiles. The *P. salivibrio* genome was compared to the compendium of resources for dealing with osmotic stress discussed for *Synechococcus* [44] and other systems as represented in the panel of representative genomes (Additional file 2: Table S2, osmotic stress). At the most basic level, in all bacteria Na^+^ is actively exported and K^+^ is actively imported to balance osmotic stress. *P. salivibrio* does not have the major sodium exporters (NhaA, NhaB) found in *E. coli* [45], but instead has another common multisubunit Na^+^ exporter (Additional file 10: Table S6, osmotic stress). *P. salivibrio* also has one of a selection of common K^+^ importers. Among the representative stains, each appears to have a relatively minimum selection among these common transporters with little indication of a streamlining effect, although the vertical indexes in *P. salivibrio* suggest that horizontal transfer is a common theme in this set of genes. However, *P. salivibrio* has a kefB-like transporter variously described as a K^+^ or Na^+^ exporter, and which fluctuates with a number of paralogs from 0 to 7 in the representative genomes with a significant streamlining effect. This would imply some auxiliary function(s) not essential to osmotic regulation for the kefB family.

For low salt stress, *P. salivibrio* has two mechanosensitive channels thought to release salt ions relatively nonselectively. There is a similar gene content across the representative panel. For high salt stress, there are a variety of osmolites accumulated by bacteria. *P. salivibrio* appears to have pathways to synthesize and degrade one of these, trehalose (Additional file 10: Table S6, osmotic stress). Among the ABC transporters, *P. salivibrio* has one, C3B54_11782–5, which appears to be orthologous to a documented H37Rv glycine-betaine importer. Glycine-betaine (trimethylglycine) is a common osmolite, although *P. salivibrio* does not appear to have the enzymology to synthesize or catabolize it. Finally other amino acids, or amino acid derivatives are commonly used for osmolites. *P. salivibrio* appears to have little enzymology to catabolize the more complex amino acids, with the exception of proline (see below). If proline were used as an osmolite, that would mandate pathways to both catabolize it and synthesize it.

### Interactions with toxic substances

*P. salivibrio* was found to be resistant to gentamicin, polymyxin B, and mitomycin C [1]. It was sensitive to antibiotics that work externally to the membrane (beta-lactams, vancomycin, and bacitracin) [46, 47]. The genome contained no beta lactamase genes or vancomycin resistance operon. However, C3B54_11952 is a gene often grouped with vancomycin resistance determinants and thought to confer low-level resistance to teicoplanin (a glycopeptide antibiotic). Bacitracin resistance is associated with polyrhamnose-containing cell walls [47], which *P. salivibrio* has (see below). But apparently the specific requirements for resistance are not met. Two intrinsic resistance systems could be identified, that could be involved in resistance to gentamicin, polymyxin B, mitomycin C and other toxic substances. 1) Some of the encoded toxin/antitoxin systems, specifically the Hip family, are known for sensing drug-induced stress and switching the bacteria to a persistent non-growing state that enhances survival of some challenges [48]. 2) There is an array of 30 exporters distributed in a wide number of classes and subclasses (Additional file 2: Table S2, exporters; Additional file 10: Table S6, exporters). Although some of these have non-defensive functions in exporting cell wall components or exporting metabolic waste, most are known by characterization of homologs as broad spectrum exporters providing multidrug resistance or relieving toxic overloads of amino acids or other metabolites. We presume those toxic overloads are aberrant states also induced by exogenous toxins. Although these systems are usually discussed due to their interference in the treatment of pathogens with high dose antibiotics, they have their origins in the biological warfare conducted between one microorganism and another in natural environments. *P. salivibrio* encodes one macromycin-like peptide of its own (C3B54_1176), and it would be difficult to exclude that it synthesizes other toxins.

HMM models were identified or constructed (as described in Methods) to represent the various families and subfamilies of exporters and used to count their incidence in the panel of representative genomes (Additional file 10: Table S6, exporters). A streamlining effect was observed similar to other families tabulated. *P. salivibrio* with 33 tabulated export genes was low compared to large genome representatives (range 40–88), below average relative to non-streamlined Microbacteriaceae (range 23–46), and above average relative to other streamlined genomes (range 15–35). The occurrence of a streamlining effect on defensive genes complicates the model for how streamlining occurs. Streamlining is not confined to dropping catabolic pathways for nutrients that are missing in the environment.

Arsenate resistance is provided by arsenate reductase (C3B54_111423) and an arsenite efflux pump (C3B54_11498). There are two members of the arsR transcription factor family (C3B54_11292, C3B54_11462), known for sensing arsinate and/or heavy metals and inducing detoxification systems. *P. salivibrio* is resistant to 0.5 mM arsenate (Additional file 1: Table S1).

*P. salivibrio* was resistant to 0.5 mM copper (Additional file 1: Table S1). Some elements of the resistance system are C3B54_11203 (CopZ, Cu^+^ cytoplasmic chaperon), C3B54_11204 (CopA, Cu^+^ exporter), C3B54_11412 (divalent ion transporter, probably exports Cu^2+^), C3B54_11118, C3B54_11724, C3B54_11746, C3B54_111704 (all copper binding proteins and chaperones).

### Hemolysins

Five *P. salivibrio* proteins were labeled hemolysins by automated annotation. By our more extensive family search, they are members of wide spread bacterial membrane protein families, typically involved in ion transport. To be an actual hemolysin, there has to be a mechanism to excrete the protein and insert it into a target cell membrane. No such mechanism was apparent in our examination of this genome. *P. salivibrio* was found not to conduct hemolysis by direct assay (Additional file 1: Table S1). The genes are labeled “hemolysin-related” in our annotated GenBank file.

### Carbohydrate utilization

*P. salivibrio* grows on glucose [1]. An ROK glucokinase (C3B54_11963) is identified homologous to the enzyme that functions to begin metabolism of glucose in *Streptomyces coelicolor* [49]. Members of this family exert transcriptional regulation coupled to their phosphorylating activity. The *P. salivibrio* enzyme lacks the N-terminal DNA binding domain often associated with this family, but the *S. coelicolor* homolog also lacks that domain but still exerts carbon catabolite repression. The mechanism is unknown, but does not involve cAMP. Similarly, *P. salivibrio* does not encode a catabolite repression protein, or an adenylylcyclase. The transporter for glucose in *P. salivibrio* is unknown. In actinobacteria, two glucose transporters are known, an MFS (Major Facilitator Superfamily) transporter, and a PTS (phosphotransferase system) transporter [50]. Although *P. salivibrio* encodes a PTS system and numerous MFS transporters, the glucose-specific versions of these transporters do not appear to be present. Of the representative genomes, the lack of those two systems is also found in *Yonghaparkia*, *Microcella*, MedAcidi-G1, *Rhodoluna*, and acMicro-4. Therefore, there may be a strategy for importing glucose in streamlined and small genome Actinobacteria that is not reflected in the literature. If the PTS system transported glucose, there would be no need for a glucokinase. Any of the other ABC or MFS transporters may have been modified to transport glucose as its primary substrate. Alternatively, modifying transporters to work with broader specificity might be a strategy for economizing on gene content.

The sole PTS system is in an operon (C3B54_111147–C3B54_111140) which includes a mannitol-1-phosphate dehydrogenase and a fructose-1-phosphate kinase. This should be all the enzymology necessary to import either mannitol or fructose and convert them to glycolytic intermediates. Mannitol is rich in coastal seawater because brown algae accumulates it [51]. Jang et al. [1] report growth on mannitol as sole carbon source by *P. salivibrio,* but it does not utilize fructose. There is a galactokinase (C3B54_111724), but *P. salivibrio* does not grow on galactose, and the pathway incorporating the galactokinase is unclear. There is no invertase, no lactose operon, no rhamnose operon, and no arabinose operon.

*P. salivibrio* encodes 7 CUT2 class ABC carbohydrate transporters (Additional file 2: Table S2, ABC). This class generally imports monosaccharides, including derivatives such as ribonucleosides. It also encodes 3 CUT1 class ABC carbohydrate transporters, which generally import oligosaccharides. By comparison to the other strains tabulated, *P. salivibrio* has more ABC carbohydrate transporters than the other streamlined bacteria, but fewer than most of the non-streamlined members of Microbacteriaceae (Additional file 10: Table S6, ABC). In its lineage, *P. salivibrio* has fewer carbohydrate transporters than *Yonghaparkia* and *Microcella*, but more than its sister streamlined acMicro-4. However, it would be an error to think of this as a progressive loss of an ancestral set of transporters. None of these transporters fall in the set of vertically descended genes. Only one of them is shared as a close homolog with acMicro-4 and none with *Yonghaparkia* and *Microcella*. By BlastP analysis, all of these have closest relatives elsewhere in Actinobacteria or even outside of Actinobacteria. These are horizontally acquired genes, all but one of which was acquired after the split from acMicro-4 ~ 0.8 Gya, and hence after the main streamlining transition. Rather than a progressive loss of genes, this distribution reflects the ongoing acquisition of genes most suitable to the current ecological niches. In that regard, *P. salivibrio* contains a higher number of ABC carbohydrate transporters than the other streamlined bacteria indicating that its coastal environment provides more diverse carbohydrate resources than experienced by the other oligotrophic bacteria. However, it does follow the trend with other planktonic bacteria of having more monosaccharide (CUT2) transporters than oligosaccharide (CUT1) transporters, as opposed to non-planktonic bacteria which tend to be loaded with oligosaccharide transporters. Hence, the *P. salivibrio* genome gives elegant confirmation that in its adaptation to coastal waters it exploits dissolved nutrients rather than adhering to biofilm or particles where disintegrating cell wall is available as a food source.

*P. salivibrio* does not grow on xylose, ribose, or *N*-acetylglucosamine (Additional file 1: Table S1). The bioinformatics analysis does not add to those observations. It is not possible to discriminate what substrates the transporters can transport, and there are enough enzymes not assigned to any specific pathway that the metabolism of a nutrient if it got in could not be excluded.

The pathways for oxidizing glucose were easily identifiable and exhibited a relatively high degree of vertical descent (Additional file 2: Table S2, carbo paths).

### Nitrogen and amino acid metabolism

The genes encoding complete pathways for synthesis of all of the amino acids (although see cys and met below) were identified in *P. salivibrio* (Additional file 2: Table S2, AA paths). Most of the enzymes appear to have descended from Microbacteriaeae ancestors, and to have orthologs in H37Rv. Ammonium assimilation is through glutamine synthase. There is no glutamate dehydrogenase, which is an energetically preferred pathway for ammonium assimilation when availability is high [52]. The lack of a glutamate dehydrogenase is shared with streamlined strains MedAcidi-G1, acMicro-4, *Prochlorococcus*, *Rhodoluna*, as well as with H37Rv and other Microbacteriaceae: *Clavibacter* and *Rathayibacter* (Additional file 10: Table S6, AA enzymes). The regulatory circuit for controlling ammonium assimilation is also identified (Additional file 2: Table S2, AA paths). The ammonium assimilation genes are also derived from Microbacteriaceae ancestors with the exception that the PII ammonium import regulator has been acquired (presumably replaced) recently.

The method of provision of sulfur for synthesis of cys and met is unclear. Both pathways have enzymes that derive sulfur from H_2_S. *P. salivibrio* encodes a sulfate assimilation pathway (Additional file 2: Table S2, misc. paths), however, no gene for 3′-phosphoadenosine-5′-phosphosulfate (PAPS) reductase, or sulfite reductase are detected, which would provide the H_2_S required for de novo cys and met synthesis. The sulfate assimilation pathway was recently acquired along with a number of PAPS-dependent sulfotransferases presumably for cell wall modification. The other Microbacteriaceae representative genomes lack the sulfur assimilation pathway. Marine bacteria often prefer exogenous reduced sulfur compounds such a dimethylsulfoniopropionate over sulfate as a sulfur source for cys and met synthesis [53]. The presence of such a path in *P. salivibrio* is unclear. Informatically, there is a pathway to synthesize met with sulfur from cys, but in culture the bacteria did not grow without supplying both met and cys (Additional file 1: Table S1).

### Amino acid uptake

*P. salivibrio* has ABC importers for both hydophilic and hydrophobic amino acids, as well as for peptides (Additional file 2: Table S2, ABC; Additional file 10: Table S6, transporters). The number of importers is minimal, but we believe sufficient to acquire all available amino acids from the environment. A suitable array of aminopeptidases is available to reduce peptides to amino acids. At least one of the peptide transporters is presumed to be tasked with recycling cell wall components. Whereas most streamlined bacteria do not secrete proteases [7], *P. salivibrio* produces a subtilisin (C3B54_11518). Such proteases in Actinobacteria are usually transported through the cell wall and released by type VII secretion systems [54], however, the *P. salivibrio* has no type VII systems. Instead the *P. salivibrio* gene encodes a signal peptide and possibly a membrane anchor. We presume the function of the enzyme is to generate amino acids and peptides for nutrition purposes, and that the revision in secretion strategy is to keep it from diffusing away from the cell in its planktonic lifestyle.

### Amino acid catabolism

*P. salivibrio* was able to grow in culture on 3 mM apiece of each amino acid with no additional carbon source. Testing for carbon source utilization was done in 0.4% yeast extract (0.2–1 mM apiece of amino acids), in which growth was minimal without carbon source supplementation [1]. In seawater, dissolved amino acid concentrations range from < 1 to 50 nM, with gly, ala, glu, and asp being the most abundant and accounting for about 60% of the total [55]. In coastal water, there is variation with maximum concentrations about 5 times higher [56]. These environmental concentrations would not support rapid growth according to the culturing experiments, but the growth rates in nature are presumed to be much lower. We made a survey of what catabolic amino acid pathways appeared to be encoded in the genome (Additional file 10: Table S6, AA enzymes).

*P. salivibrio* does not have a glutamate dehydrogenase. Growth on amino acids usually is accompanied by collecting the nitrogen by transamination on glutamate so that it can be eliminated as ammonia by this enzyme. However, *P. salivibrio* does have aspartate ammonia lyase and alanine dehydrogenase which could, in principle, be used for a similar purpose. *P. salivibrio* has only 5 members of the transaminase family, of which one is not a transaminase. The remaining four transaminases must be relatively promiscuous to handle the required load for amino acid biosynthesis, and at least those activities must be available for catabolism. Hence there would appear to be fairly direct pathways for catabolism of ala, glu, and asp.

Catabolism of gly is of particular interest since it is the most abundant of the environmental amino acids. Glycine can be converted to serine, but there is no serine dehydratase to directly convert serine to pyruvate. Transamination of serine would produce hydroxypyruvate. If one of the paralogs of the phosphopyruvate reductase encoded in the *P. salivibrio* genome has hydroxypyruvate reductase activity, then a pathway to catabolize gly through glycerate-3-P is open as proposed in Additional file 2: Table S2, AA paths. Among the more complex amino acids, there appears to be no enzymology at all for breakdown of his, tyr, phe, trp, arg, or met. There is sporadic presence of these pathways in other Microbactericeae (Additional file 10: Table S6, AA enzymes), so there is some streamlining effect for amino acid catabolism pathways. These amino acids are present in very low amounts in sea water. There is an asparaginase. However, asparaginase is necessary for use of imported asn in translation. This is because *P. salivibrio* (and all the representative genomes) do not encode an asn-tRNA synthetase. Instead they charge tRNA_Asn_ with Asp, and then amidate the asp. However, since there is a catabolic enzyme for asp, asn should also be catabolizable. There is a branched oxo-acid dehydrogenase complex that would convert val, leu, and ile to their branched acyl-CoA derivatives. However, the pathway to catabolize these molecules further is not apparent. Further catabolism of branched acyl-CoA (as well as of propionyl-CoA from degradation of odd number fatty acids) uses paralogs of acetyl-CoA carboxylase and enoyl-CoA hydratase. There are only the minimal members of these enzymes required to carry out other essential functions. In bacteria, the breakdown of val, ile, leu to branched acyl-CoA has an ulterior purpose. These molecules can be extended to branched chain fatty acids, which are common in bacterial membranes as a means of increasing fluidity [57]. All of the major fatty acids of *P. salivibrio* are branched [1]. This may be an additional factor for survival in cold water. There is a clear catabolic pathway for proline. As mentioned above, we suspect that proline may be used as an osmolite, which would require regulated pathways to increase and decrease its cellular concentration.

*P. salivibrio* encodes gluconeogenic enzymes (Additional file 2: Table S2, carbo paths). There is a pyruvate water dikinase which directly converts pyruvate to phosphoenolpyruvate. The presence of that enzyme suggests that gluconeogenesis does draw carbon from alanine through pyruvate. *P. salivibrio* also encodes phosphoenolpyruvate carboxylase to convert oxaloacetate to phosphoenolpyruvate. That provides an entry point for citric acid cycle intermediates into gluconeogenesis as would arise from transamination (or oxidative deamination) of asp or glu.

### Fatty acid synthesis

Many bacteria have a fatty acid synthase consisting of a large multifunctional polypeptide dedicated to synthesizing C16 fatty acid, and additional enzymes distributed across multiple polypeptides that can extend the C16 fatty acid to longer lengths. H37Rv has several paralogs of the multifunctional fatty acid synthase thought to synthesize a variety of polyketides. *P. salivibrio* does not encode a multifunctional fatty acid synthase, but does encode the distributed system (Additional file 2: Table S2, lipids). The number of systems encoded in a genome can be counted by the number of acyl carrier protein (ACP) domains, either as small standalone protein for distributed systems, or as part of a large polyprotein (Additional file 10: Table S6, lipids). *P. salivibrio* encodes one small ACP (C3B54_11898), which is typical of the streamlined representative genomes. This contrasts with 1–5 in Mycobacteriaceae in general, and up to 59 in the large Streptomyces genome. The polyketide synthetic systems make a wide variety of molecules, including antibiotics [58]. The absence of the polyketide synthases may constitute the strongest streamlining effect of any function we categorized in *P. salivibrio*.

The distributed fatty acid synthesis system employs a type III 3-oxoacyl-ACP synthase which initiates the synthesis by condensing a malonyl-ACP with a primer, which may be either acetyl-CoA, or a branched chain acyl-CoA derived from amino acid metabolism (see above). Correspondingly there are two versions of this enzyme (C3B54_11899, C3B54_11450). In subsequent cycles, a type II 3-oxoacyl-ACP synthase (C3B54_11897) extends the existing acyl-ACP by condensing with another malonyl-ACP. Of the other steps in the synthetic cycle, 3-oxoacyl-ACP reductase, and acyl dehydratase are identified. The required 2-enoyl-ACP reductase has not been identified in the *P. salivibrio* sequence, although it is generally expected to be of the short chain dehydrogenase family of which there are 14 paralogs encoded in *P. salivibrio*. Closely related sequences to any characterized enoyl-ACP hydratase are not present. This situation is generally true through the representative Mycobacteriaceae genomes (data not known). Similarly the thioesterase required to release the finished product from ACP is not identified.

### Phospholipid synthesis

The major polar lipids of *P. salivibrio* were reported to be phosphatidylglycerol and diphosphatidylglycerol [1]. A survey of reported lipid content of numerous members of Microbacteriaceae revealed these same two major phospholipids, and no mention of phosphatidylserine, phosphatidylethanolamine, phosphatidylcholine, or phosphatidylinositol (not shown). There are, however, often reports of minor unidentified phospholipids and glycolipids. Informatically, the pathway to produce the major phospholipids, phosphatidylglycerol and diphosphatidylglycerol, was identified (Additional file 2: Table S2, lipids). No candidates for enzymes to make phosphatidylethanolamine, phosphatidylcholine, or phosphatidylinositol were identified. The key enzyme to add glycerol to phosphatidic acid, was present in three paralogs, C3B54_11955, C3B54_111393, C3B54_11702. Two of these might transfer alcohols other than glycerol to make other minor phospholipid species, although none of them was similar to any established serine transferase.

### Fatty acid catabolism (Beta oxidation)

*P. salivibrio* encodes a beta oxidation system (Additional file 2: Table S2, lipids). Oddly, although many Microbacteriaceae have such a system, *P. salivibrio* seems to have acquired its system by horizontal transfer. Its closest relative, acMicro-4, appears to have no beta oxidation system at all, so it may be true that beta oxidation was lost in the *P. salivibrio* lineage, and then reacquired. There are no paralogs of the 5 enzymes within the *P. salivibrio* genome, with the exception that the naphthoate synthase involved in menaquinone production is a paralog of the enoyl-CoA hydratase. In contrast, most Microbacteriaceae have multiple paralogs of the various enzymes involved, and the large genome bacteria can have over a dozen paralogs (Additional file 10: Table S6, lipids). Hence, *P. salivibrio* may be considered to have a minimal beta oxidation system.

### Other pathways

*P. salivibrio* encodes all the typical enzymes for synthesis of nucleotides, including salvage enzymes for hypoxanthine/guanine and thymine. Pathways to synthesize pyridoxal phosphate, folate, CoA, and riboflavin were found, as well as a pathway to scavenge nicotinamide to NAD and NADP (Additional file 2: Table S2, misc. paths). No facility to use molybdopterin or cobalamin was detected.

### Phosphate metabolism

*P*. *salivibrio* imports phosphate with a high-affinity ABC Pst family transporter (C3B54_11275–8). It has no low affinity PitA MFS family transporter, a situation in common with most streamlined bacteria (not shown). The global phosphate regulatory system described [59] is present: Pst, two component PhoR/B (C3B54_11432–3), and PhoU (C3B54_111707). 5′-nucleotidase and alkaline phosphatase, two enzymes thought to be important for acquiring phosphate from organic phosphate in marine bacteria [60], were not encoded in *P. salivibrio*, and were not found in any of the representative streamlined genomes.

### Cell wall

The peptidoglycan synthesis genes of *P. salivibrio* have been identified (Additional file 2: Table S2, cell wall). They are mostly typical of other Microbacteriaceae and H37Rv. There are three crosslinking transpeptidases (C3B54_11386, C3B54_11194, C3B54_11975), all of the D,D-transpeptidase variety. That is, crosslinks are directly from one pentapeptide side chain to another, with no intervening pentaglycine bridge. Correspondingly, no enzymology for synthesis of a pentaglycine crossbridge was in evidence. There were three periplasmic enzymes encoded of a type known to acetylate peptidoglycan and confer lysozyme resistance. Each of those three was obtained recently by horizontal transfer.

In non-Gram negative bacteria, there are typically complex carbohydrate or lipocarbohydrate structures attached to the peptidoglycan. These are usually composed of an arabinoglycan core with mycolic acid attached in Mycobacteria, a teichoic acid core with complex oligosaccharides attached in Gram positive bacteria, and rarely a polyrhamnose core with complex oligosaccharides attached [61]. Synthesis of mycolic acid, at least in *Mycobaterium tuberculosis*, requires a polyketide synthase for one of its steps. *P. salivibrio* has no polyketide synthases, and so probably carries no mycolic acid. There was also no enzymology detected to make arabinoglycan. At least two genes required to make teichoic acid (TagB and D) were missing. Hence, we believe that the major carbohydrate portion of the *P. salivibrio* cell is based on a polyrhamnose core. The proposed pathway is given (Additional file 2: Table S2, cell wall). Marker genes for teichoic acid, rhamnose, and arabinoglycan synthesis were tabulated in the representative genomes (Additional file 10: Table S6, cell wall). Unfortunately, rhamnose is used within the other types of cores, so the marker gene is not definitive for its use as the oligosaccharide core. However, it seems that Microbacteriaceae usually use teichoic acid, but MedAcidi-G1 and *Rathayibacter* do not seem to have an alternative, so they may also use polyrhamnose.

There is a large collection of encoded glycosyltransferases, most of which presumably function to add a variety of residues to the peptidoglycan carbohydrate. Summing over several families of these (Additional file 10: Table S6, cell wall) revealed that *P. salivibro* with 28 glycosyltransferases has more than the other streamlined bacteria (range 8–18). It was in the low end of the range for non-streamlined bacteria (range 24–45). The vertical indexes of the glycosyltransferases indicated a high incidence of horizontal transfer, corresponding to relatively rapid revision in the cell wall over evolutionary time. *P. salivibrio* appeared to add sugars of types not seen elsewhere in the representative panel. These were fucose, sialic acid, and keto-deoxyoctulosonate (KDO). KDO, which is part of the Gram negative outer envelope core, is rare outside of Gram negatives. The closest BlastP matches of the *P. salivibrio* KDO synthesis genes were to incidences found outside of Gram negative bacteria (data not shown).

### Other paralogs

There are numbers of protein families, e.g. short chain dehydrogenases, usually present in multiple copies with only a few assigned to some specific essential role. *P. salivibrio* is in the low end of the range for non-streamlined genomes, but in the high end for streamlined genomes (Additional file 10: Table S6, paralogs). But there are idiosyncratic excesses of individual families in specific streamlined genomes (e.g. enolases in *P. salivibrio*, short chain dehydrogenases in MedAcidi-G1, epimerases in *Prochlorococcus* and *Pelagibacter*) illustrating that specific metabolic needs often override the overall streamlining pressure.

### Pseudogenes and selfish genes

It is commonly reported that streamlined genomes have fewer pseudogenes. We find a range of 5 to 359 pseudogenes annotated on the representative genomes (data not shown), but suspect that differences in annotation practices probably obscures any meaningful trends if present. On the other hand, selfish genes of two kinds can be objectively counted: integrated phage genes, and insertion elements. Searching for phage terminase genes as a means of counting integrated phages revealed a few in the representative genomes, but none in streamlined genomes (Additional file 10: Table S6, selfish). There was not a single gene in *P. salivibrio* that we could confirm was of phage origin. The nine IS elements located in *P. salivibrio* (Additional file 11: Table S7) is a relatively low number compared to those representative genomes that have them, but we found only one IS element (searching for transposase) in all other streamlined representative genomes combined (Additional file 10: Table S6, selfish).

### Codon preference

Finally, we explored codon preference in the *P. salivibrio* genome. Codon preference is enforced by very small selective advantages (per codon) of using more abundant tRNAs for proteins required to be expressed more abundantly [62]. We thought that the codon preference system might disappear under streamlining, either because slow growing bacteria were under less pressure to express any protein rapidly, or because the small effective population size usually attributed to such bacteria undercut the impact of very small selective pressures. However, in *P. salivibrio*, we found that proteins well known to be abundantly expressed, such as ribosomal subunits, were strongly correlated with their rank on the principle axis of the correlation analysis conducted by codonW (Additional file 12: Table S8). The position of all ribosomal subunits on the list was tabulated yielding an average position on the list at the top 7th percentile. That shows that codon preference related to translational efficiency has not collapsed in *P. salivibrio*. Screening the representative large genomes with the set of preferred codons determined from *P. salivibrio* (Additional file 13: Table S9) produced a similar ranking of ribosomal protein genes (data not shown). So the Actinobacteria in general have the same codon preferences related to translation. That assay is qualitative, so it does not clearly establish if the strength of selection for those codons is the same in the different genomes. The preferred codons end in G or C for many but not all amino acids. So selection for translational efficiency could conceivably raise the overall %G + C of the genome. We were interested if there could be any coupling between intensity of selection for translational efficiency and the decreasing %G + C seen associated with streamlining (Additional file 6: Table S4). That possibility seems to be eliminated by the observation that the overall %G + C of the genes on the top of the sorted list was essentially the same as the genes on the bottom of the sorted list (data not shown). So, there is not enough preference for codons ending in G or C in the translationally preferred codons to affect overall base composition of the genome.

## Conclusions

*P. salivibrio* is a photoheterotrophic streamlined bacterium, even though it grows in a non-oligotrophic coastal marine zone. The pathway through which this bacterium has adapted to this zone may have involved passage through a more traditional streamlined oligotrophic state followed by reacquisition of some functions. This idea is supported by common ancestry with the streamlined acMicro-4 freshwater Actinobacterial lineage roughly at the end of the snowball Earth glaciations. In many categories of genes, *P. salivibrio* has fewer than Microbacteriaceae in general, but more than the average streamlined bacteria. Two functional groups appear to not be streamlined in their numbers: toxin/antitoxin systems, and cell wall glycosyltransferases. Most of the genes in those two categories have been acquired horizontally. Approximately half of the genome appears to have been acquired or replaced by horizontal transfer since the Microbacteriaceae radiation, supplying ample opportunity for fine tuning of the phenotype. Functions related to oxidative or osmotic stress, which intuitively might require adjustment in adaptation to the coastal environment are a mixture of vertically descended and horizontally transferred (possibly replaced) components, which may have provided a means to reoptimize them. On the other hand, the bacteriorhodopsin appears to have descended from Microbacteriaceae ancestors, leaving open the possibility that this lineage maintained its capacity for photoheterotrophy through the snowball Earth glaciations. The most fundamental question, how can an apparently slow growing bacterium persist in an environment that also supports fast growing bacteria, remains a challenge. The larger number of toxin/antitoxin systems may be a clue, perhaps signaling a strategy of occupying micro-niches within the environment where transient nutrient deprivation or other transient stresses are particularly severe.

## Methods

### Genome sequencing

Genome sequencing of *Pontimonas salivibrio* CL-TW6^T^ was performed by three sequencing platforms: 454, Illumina, and PacBio. For the 454 and Illumina sequencing, genomic DNA of the strain was extracted by MG Genomic DNA Purification Kit (Doctor Protein) for cells in late-stationary growth phase cultivated on marine agar 2216 (MA; Difco) at 30 °C for 14 d. A sequencing library was prepared with GS DNA Library Preparation Kit (Roche) and sequenced on 1/8 region of PicoTiterPlate device in the 454 GS FLX+ system (Roche) by Macrogen Co. (Korea). Two sequencing libraries were prepared with Truseq PCR-free Sample Preparation Kit (Illumina; fragment size of ca. 350 bp) and Nextera XT DNA Sample Preparation Kit (Illumina; insert size of ca. 5 kb) according to the manufacturer’s instructions to obtain paired-end and mate-pair reads, respectively, and then sequenced on Illumina HiSeq 2000 platform using paired-end 2 × 100 bp chemistry by Macrogen Co. (Korea). For the PacBio sequencing, genomic DNA was extracted by DNeasy Blood & Tissue Kit (Qiagen) for cells in late-exponential growth phase cultivated on MA at 30 °C for 9 d. A sequencing library was constructed with SMRTbell Template Prep Kit following manufacturer’s instruction (Pacific Biosciences; fragment size of > 20 kb) and sequenced with PacBio RS II (Pacific Biosciences) platform using 2 single molecule real time (SMRT) chemistry cells and C4 chemistry by DNA Link Co. (Korea).

### Assembly

Paired end Illumina reads, 454 reads, and regions of contiguous assembly (contigs) were returned by Macrogen. The contigs were terminated by IS elements, and paired end reads from the flanking regions were used to sew the contigs together into a circular chromosome. Unique polymorphisms within the IS elements were similarly subjected to paired end analysis to make sure that the exact sequence of each IS element was correctly assigned to each map position. The sequence of each uninserted site was constructed and searched against the reads, revealing that one of the IS elements occupied its insertion site at only 85% allele frequency. There was one contig left over composed of reads represented at only 2% the stoichiometry of the bacterial genome. Further analysis indicated that this is a phage genome, designated now as phiPsal1.

De novo assembly of PacBio data was performed using the hierarchical genome assembly process workflow of the SMRT Analysis (ver. 2.3.0; Pacific Biosciences). In parallel, de novo hybrid assembly was performed using SPAdes (ver. 3.7.1) [63] from Illumina reads and PacBio filtered subreads (processed from SMRT Analysis). For the two assembled sequences, genome alignment was made using Mauve (ver. 2.3.1) [64] and BlastN. Different regions of the alignment were inspected and manually corrected on the basis of read mapping with Illumina, PacBio (i.e. filtered subreads) and 454 reads using Geneious (ver. 9.1.5) [65]. The only meaningful differences between the Illumina/454 data and the PacBio data are: one IS in the Illumina/454 data that was at a partially occupied site was missing in the PacBio data. The phiPsal1 phage is missing in the PacBio data. There is one region of simple sequence that is of wildly varying length among different reads, whether 454, Illumina, or PacBio. That sequence was arbitrarily set to C_15_.

### Annotation

Initial genome annotation was performed by both Prokka (ver. 1.11) [66] and the IMG/ER annotation pipeline [67]. Initial identification of non-protein encoding genes was done with Prokka with an option of searching non-coding (nc) RNAs. CRISPRs were searched by both Prokka and CRISPRFinder [68], but none were found. Start codon positions were refined using GeneMark [69], a local implementation of GBrowse [70] to highlight overlapping frames, and BlastP searches of NCBI’s nr, and env_nr as well as the TARA [71] and MG-RAST [72] metagenomic databases. In the BlastP searches, the frames were extended upstream to reveal incidences of protein similarity extending upstream of the initially assigned start codon. All regions between ORFs were then searched with tblastx against NCBI nr, finding one additional gene, and one candidate for a fragmented pseudogene. All noncoding sequence was then searched against Rfam [73], finding a few additional RNA motifs.

The initial suggested gene annotations were compared to the results of HMM searches at the hhpred web site [74], which at the time included models derived from PDB [75], Pfam [76], COGS [77], TIGRFAM [78], and NCBI CDD [79], to find a list of plausible alternative annotations for each gene. To search for close homologs that had been biochemically characterized, a customized blast library was prepared by importing the GenBank files of all bacterial genomes marked as “complete genome” and extracting protein sequence with a definition line that included all available annotation (tags for notes, experiments, etc). Searching the *P. salivibrio* genes against that library often provided links to papers having established the function of a close blast match. Those nearly always came from the heavily annotated entry GenBank file for *Mycobacterium tuberculosis* H37Rv. Once having established an ortholog in H37Rv, we also used the remarkably useful and unique tendency of authors studying *M. tuberculosis* to put locus tags in the abstracts of their publications to find additional papers clarifying the functions of individual genes. We have placed PubMed numbers in our GenBank entry to capture this information. For studying horizontal and vertical descent in linkage, we included the nucleotide accession and coordinates on the definition line. Analysis of the rhodopsin gene used the MicRhoDE server [80]. Signal sequences were documented using SignalP [81]. Transmembrane helixes were documented with TMHMM [82].

### Comparison among genomes

BlastP in the NCBI nr library was conducted and taxonomic groups found near the top of the BlastP listings were tabulated. All taxa found disproportionately were found to already be in a custom-curated library of all bacterial genomes that we had assembled from 2015, except for a genome of unclear finished status from *Yonghaparkia*, a *Microcella alkaliphila* genome, and a metagenomic assembly named acMicro-4. These were added to the library. In choosing more divergent representative strains for phylogenetic analysis, we chose three taxa not already in that library (*Pelagibacter ubique*, *Prochlorococcus marinus* MED4, and MedAcidi-G1) and added them also. Accessions and references for genomes used are given in Table 1. Additional file 14 includes a script that can be used to generate these complete genome libraries with extended definition lines. However, additional curation steps are necessary because NCBI is inconsistent in the application of the definition “complete genome”, and also to remove redundancy caused by most but not all GenBank entries having a duplicate RefSeq entry. Whereas we have checked our work in a more recent and larger library, due to the complexities of producing a well curated library with one and only one copy of every gene from complete genomes only, we confine our reporting to results from the supplemented 2015 complete genome library.

In the complete genome library, there were 10 Microbacteriaceae genomes. Core gene analysis consisted of identifying the number of CLTW6 genes finding a BlastP match in all 10 of those genomes. Construction of neighbor joining trees with PAUP [83] among core genes indicated ample incongruencies, hence a selected mutually congruent set was catenated for phylogenomic tree analysis. The phylogenomic tree was further restricted to a subset of genes that was deemed particularly likely to uncover a more recent common ancestor to just *P. salivibrio*, *Microcella*, *Yonghaparkia*, and acMicro-4 based on the BlastP ranking of those matches as described in the main text. One of these congruent genes (DnaE) was used for mapping of single gene metagenomic data. Both phylogenomic and DnaE tree were calculated in the form of a time tree by Bayesian inference using MrBayes [84] using the relaxed clock option with the default independent gamma rate parameter.

In order to reduce the core to a congruent core, we tabulated those core genes for which the top 10 matches were the 10 Microbactericeae genomes. This congruency constraint will remove genes from the core for which the *P. salivibrio* version was introduced by horizontal gene transfer from a non-Microbacteriaceae source causing that far away taxon to have more similarity by BlastP than the Microbacteriaceae matches. In this counting process, as explained in the text, we generated a parameter which we call “vertical index” indicating how many of the top 10 matches were from Microbacteriaceae, and hence how close the gene was to a core gene exhibiting core-like vertical descent from the Microbacteriaceae common ancestor. A script to aid in counting the vertical index is given in Additional file 14.

To understand relationships among representative genomes with respect to numbers of paralogs and presence or absence of specific pathways required counting paralogs in the different GenBank files. We were at first frustrated by the inconsistency in annotation practices across the selection of genomes. To remove reliance on other authors’ annotations we first created genome-specific fasta libraries for each genome. We imported the Pfam28.0 library and the hmmer [85] programming system and used hmmscan to find Pfam models representing specific homology groups of interest. Then we scripted an operation to search each genome with hmmsearch and report the number of family members detected. In each case, the threshold of the search was adjusted based on observing the e-value at which obviously misleading matches were arising in our genome or in H37Rv. In many cases there simply is not a Pfam model of useful discriminatory power to conduct this operation. Discrimination was judged by scoring test genomes and asking if there was a similarity threshold that cleanly separates the class of paralogs we wished to tabulate for other genes that are related but known to be functionally distinct (for example, helicases versus ATPases of other kinds, or transposases versus other genes with helix-turn-helix motifs). Test genomes were initially our *Pontimonas* genome and H37Rv, where extensive human curation has been applied to identify related genes that are better matched to some other functional specification. If additional test genomes were needed, for example in the case of constructing new HMMs, others of the representative genomes were used and the matched genes were scored through NCBI CDD to identify related but functionally distinct genes.

If necessary a discriminatory HMM was constructed using the UCSC sequence alignment and modeling system [86]. The basic procedure was to first align paralogs from one test genome, convert to an HMM of the Pfam format, and then score additional test genomes looking for discrimination. As necessary, more paralogs were added from those test genomes to the alignment/HMM and additional test genomes were scored until discrimination was achieved.

In some cases we utilized comparative data concerning genes matching a classification at the Transporter Classification Database (TCDB) [87]. We imported the entire TCDB database, and engineered BlastP searches of all genes in all representative genomes against that database. A script was then written to tabulate all output files of that operation to discover the numbers of incidences of a particular TCDB classification in each genome.

### Genome recruitment analysis

Genome recruitment analysis was performed by searching the MG-RAST metagenome database (as of Sept. 2016) [72] for sequences matching *P. salivibrio* 16S rRNA sequence at > 97% identity and in datasets matching the word “marine”. The presence of *P. salivibrio* 16S rRNA sequence was also explored by BlastN searches against the global ocean microbiome datasets of GOS [88] and TARA [71], and further confirmed by phylogenetic analysis with related species in Microbacteriaceae. Similarly sequences matching *P. salivibrio* DnaE were searched, and filtered by phylogenetic analysis for matching the *P. salivibrio* lineage more recently than the branch point with acMicro-4. (The acMicro-4 dataset does not have an intact 16S rRNA sequence to establish this reference point for 16S rRNA similarity). The Ocean Data View tool [89] was used to prepare a figure illustrating the geographic locations of the recovered matching sequences.

### Codon preference

Codon preferences were calculated using codonW [90]. To avoid an influence by horizontally transferred genes, the correlation analysis was carried out over genes having a vertical index of 5 or more. The correlation was carried out using the -rscu option.

## Additional files

Additional file 1: **Table S1.** Additional phenotypic traits measured for *P. salivibrio* CL-TW6^T^. (DOC 35 kb)

Additional file 2: **Table S2.** Loci from *P. salivibrio* CL-TW6^T^ grouped by pathway or functional category with vertical indexes: Loci involving light, Pathways for synthesizing isoprenoid products, Pathways of carbohydrate utilization and synthesis, Genes related to planktonic vs. biofilm lifestyle, Sigma factors and regulators, Systemic regulators in *P. salivibrio* CL-TW6^T^, Redox systems, Proteins involved in heat and cold shock, Genes related to osmotic stress, Possible exporters in *P. salivibrio* CL-TW6^T^, ABC importers, Amino acid biosynthesis and catabolism pathways, Miscellaneous paths, Cell wall synthesis, and Lipid pathways. (XLS 148 kb)

Additional file 3: **Figure S1.** Key metabolic pathways in *P. salivibrio* CL-TW6^T^. APS, adenosine-5′-phosphosulfate; DMAPP, dimethylallyl pyrophosphate; EMP, Embden-Meyerhof-Parnas; FPP, farnesyl pyrophosphate; G3P, glyceraldehyde-3-P; GGPP, geranylgeranyl pyrophoshpate; IPP, isopentenyl pyrophosphate; MEP, methylerythritol phosphate; MQ, menaquinone; PAPS, 3′-phosphoadenosine-5′-phosphosulfate; ROS, reactive oxygen species. (DOC 3698 kb)

Additional file 4: **Figure S2.** Maximum likelihood tree of 16S rRNA sequences placing representative genomes within the context of Microbacteriaceae type strains and other selected representatives. Two asterisks - complete genome available, One asterisk - incomplete genomic data available, Yellow highlight - representative genome used in this study. The tree was made with the MEGA package (Kumar et al., 2016). Only bootstrap values (expressed as percentage of 1000 replications) greater than 70% are shown at nodes. Bar, 0.05 nucleotide substitutions per site. (DOC 455 kb)

Additional file 5: **Table S3.** A pair-wise comparison of selected genomes for (a) in silico DNA-DNA hybridization (DDH), (b) average amino acid identity (AAI), (c) δ* differences (the average difference of dinucleotide relative abundance), and (d) the percentage of conserved proteins (POCP). 1, *Pontimonas salivibrio* CL-TW6^T^; 2, *Rhodoluna lacicola* MWH-Ta8^T^; 3, *Yonghaparkia* sp. Root332; 4, *Microcella alkaliphila* JAM AC0309; 5, acMicro-4. (PDF 60 kb)

Additional file 6: **Table S4.** Distribution of best BlastP matches, %GC, genome size, and numbers of tRNA and rRNA genes in representative Actinobacteria genomes. (DOC 40 kb)

Additional file 7: **Figure S3.** Phylogenomic tree. Encoded protein sequences catenated were selected from among large genes, with congruent single gene trees, and enriched with genes having BlastP results suggesting greater similarity of *P. salivibrio* with *Yonghaparkia* than to *Microbacteria*, *Clavibacter*, *Rathayibacter*, and *Leifsonia*. The computation is specifically designed to accept or reject a closer common ancestor with *Yonghaparkia*. Genes used were C3B54_11793, C3B54_11942, C3B54_11990, C3B54_111069, C3B54_111092, C3B54_111098, C3B54_111113, C3B54_111132, C3B54_111675. Time scale was determined as described in Fig. 2. (DOC 78 kb)

Additional file 8: **Table S5.** Raw vertical index numbers for all *P. salivibrio* genes. (XLS 225 kb)

Additional file 9: **Figure S4.** Distribution of genes likely derived from horizontal transfer as indicated by low vertical index. A custom built BlastP library containing one entry for each gene in completely sequenced bacterial genomes was searched with each annotated *P. salivibrio* translated gene sequence, and the number of top 10 hits mapping to a genome classified as belonging to Microbacteriaceae was plotted in genomic order. (DOC 67 kb)

Additional file 10: **Table S6.** Number of paralogs of given protein families or presence/absence of a gene marking a particular pathway or function in representative genomes: Sigma factors, Numbers of two component systems and toxin antitoxin systems in representative genomes, Redox systems, Heat and cold stress, Genes related to osmotic stress, Potential drug/toxic substance exporters, Numbers of transporters of various classes and subclasses in representative genomes, Presence/absence or number of paralogs of enzymes of amino acid metabolism in representative genomes, Numbers of paralogs associated with fatty acid synthesis and degradation in representative genomes, Cell wall, Count of paralogs in selected large families in representative genomes, and Numbers of selfish entities in representative genomes. (XLS 62 kb)

Additional file 11: **Table S7.** Insertion sequences in *P. salivibrio* CL-TW6^T^. (DOC 40 kb)

Additional file 12: **Table S8.** Top ranked genes (of 764 *P. salivibrio* genes prescreened for vertical descent) for preferred codons by codonW. (XLS 24 kb)

Additional file 13: **Table S9.** Frequency of optimal codon index of codons calculated by codonW in *P. salivibrio* CL-TW6^T^ genes. (XLS 24 kb)

Additional file 14: **Scripts**. Scripts used in this project. (TXT 13 kb)

## Abbreviations

AI-2: Autoinducer-2
ECF: Extracellular function (a classification of sigma factors)
HMM: Hidden Markov model
IS: Insertion sequence
MSF: Major Facilitator Superfamily
ORF: Open reading frame
PAPS: 3′-phosphoadenosine-5′-phosphosulfate
PTS: Phosphotransferase system
SMRT: Single molecule real time
TCDB: Transporter classification database
