# Supplementary material for: Complete genome of streamlined marine actinobacterium Pontimonas salivibrio strain CL-TW6T adapted to coastal planktonic lifestyle
Source: BMC Genomics. 2018 Aug 22;19:625. doi: 10.1186/s12864-018-5019-9 (PMC6106888; doi:10.1186/s12864-018-5019-9)
Supplement: Supplementary file 1 — Table S1. Additional phenotypic traits measured for P. salivibrio CL-TW6T. (DOC 35 kb) [file 12864_2018_5019_MOESM1_ESM.doc]

**Table S1. Additional phenotypic traits measured for *P. salivibrio* CL-TW6^T^.**

| Bacitracin^a^ | sensitive |
| --- | --- |
| Arsenic resistance^b^ | ≥ 0.5 mM |
| Copper resistance^b^ | ≥ 0.5 mM |
| H_2_O_2_ resistance^c^ | +/- at 0.5 mM; - at 1 mM |
| Hemolysis^d^ | - |
| Carbon utilization^e^ |  |
| Growth on xylose | - |
| Growth on fructose | - |
| Growth on ribose | - |
| Growth on rhamnose | - |
| Growth on N-acetylglucosamine | - |
| Growth on trehalose | - |
| Growth on amino acids^f^ |  |
| all 20 | + |
| 19 -cys | - |
| 19 -met | - |

^a^Tested by disc-diffusion with a 2 µg Bacitracin disc.

^b^Tested by growing on agar made with marine broth (Difco) supplemented with As_2_O_3_ or CuSO_4_ at 0.01, 0.05, 0.1 or 0.5 mM and culturing at 30 °C.

^c^Tested in saline Luria-Bertani (mLB) broth [10 g peptone, 5 g yeast extract, 1 L artificial seawater (Lyman and Fleming, 1940)] supplemented with H_2_O_2_ to 0, 0.01, 0.05, 0.1, 0.5, and 1.0 mM. Growth was monitored at OD_600_ at 1–3 day intervals for 2 weeks.

^d^*P. salivibrio* grew well on saline blood agar (per L) 40 g blood agar base, 50 ml sheep blood, 30 g NaCl at 30 °C. However, hemolysis was not observed to occur.

^e^Testing for growth on carbon sources was conducted in (per L) 23.6 g sea salts (Sigma), 0.05 g yeast extract, and 0.4% of the carbon source.

^f^Tested in artificial seawater (per L) 23.5 g NaCl, 5 g MgCl_2_, 4 g Na_2_SO_4_, 1.1 g CaCl_2_, 0.7 g KCl, 0.2 g NaHCO_3_, 0.1 g KBr, 0.026 g H_3_BO_3_, 0.024 g SrCl_2_, 0.003 g NaF (Lyman and Flemming, 1940), supplemented with (per L) 3 g K_2_HPO_4_, 1 g NaH_2_PO_4_, 1 g NH_4_Cl, 0.3 g MgSO_4_•7H_2_O, 1 ml of Holden's trace elements (Holden et al., 2001), 1 ml of Balch's vitamin solution (Balch and Wolfe, 1976), and brought to 3 mM with each of the indicated amino acids.

**References**

Balch WE, Wolfe RS. New approach to the cultivation of methanogenic bacteria: 2-mercaptoethanesulfonic acid (HSCoM)-dependent growth of *Methanobacterium ruminantiumin* a pressurized atmosphere. Appl Environ Microbiol. 1976;32:781–91.

Holden JF, Takai K, Summit M, Bolton S, Zyskowski J, Baross JA. Diversity among three novel groups of hyperthermophilic deep-sea *Thermococcus* species from three sites in the northeastern Pacific Ocean. FEMS Microbiol Ecol. 2001;36:51–60.

Lyman J, Fleming RH. Composition of sea water. J Mar Res. 1940;3:134–46.
