# Supplementary material for: Complete genome of streamlined marine actinobacterium Pontimonas salivibrio strain CL-TW6T adapted to coastal planktonic lifestyle
Source: BMC Genomics. 2018 Aug 22;19:625. doi: 10.1186/s12864-018-5019-9 (PMC6106888; doi:10.1186/s12864-018-5019-9)
Supplement: Supplementary file 3 — Figure S1. Key metabolic pathways in P. salivibrio CL-TW6T. APS, adenosine-5′-phosphosulfate; DMAPP, dimethylallyl pyrophosphate; EMP, Embden-Meyerhof-Parnas; FPP, farnesyl pyrophosphate; G3P, glyceraldehyde-3-P; GGPP, geranylgeranyl pyrophoshpate; IPP, isopentenyl pyrophosphate; MEP, methylerythritol phosphate; MQ, menaquinone; PAPS, 3′-phosphoadenosine-5′-phosphosulfate; ROS, reactive oxygen species. (DOC 3698 kb) [file 12864_2018_5019_MOESM3_ESM.doc]

Figure S1. Key metabolic pathways in *P. salivibrio* CL-TW6^T^. APS, adenosine-5'-phosphosulfate; DMAPP, dimethylallyl pyrophosphate; EMP, Embden-Meyerhof-Parnas; FPP, farnesyl pyrophosphate; G3P, glyceraldehyde-3-P; GGPP, geranylgeranyl pyrophoshpate; IPP, isopentenyl pyrophosphate; MEP, methylerythritol phosphate; MQ, menaquinone; PAPS, 3'-phosphoadenosine-5'-phosphosulfate; ROS, reactive oxygen species.
