# Supplementary material for: Complete genome of streamlined marine actinobacterium Pontimonas salivibrio strain CL-TW6T adapted to coastal planktonic lifestyle
Source: BMC Genomics. 2018 Aug 22;19:625. doi: 10.1186/s12864-018-5019-9 (PMC6106888; doi:10.1186/s12864-018-5019-9)
Supplement: Supplementary file 4 — Figure S2. Maximum likelihood tree of 16S rRNA sequences placing representative genomes within the context of Microbacteriaceae type strains and other selected representatives. Two asterisks - complete genome available, One asterisk - incomplete genomic data available, Yellow highlight - representative genome used in this study. The tree was made with the MEGA package (Kumar et al., 2016). Only bootstrap values (expressed as percentage of 1000 replications) greater than 70% are shown at nodes. Bar, 0.05 nucleotide substitutions per site. (DOC 455 kb) [file 12864_2018_5019_MOESM4_ESM.doc]

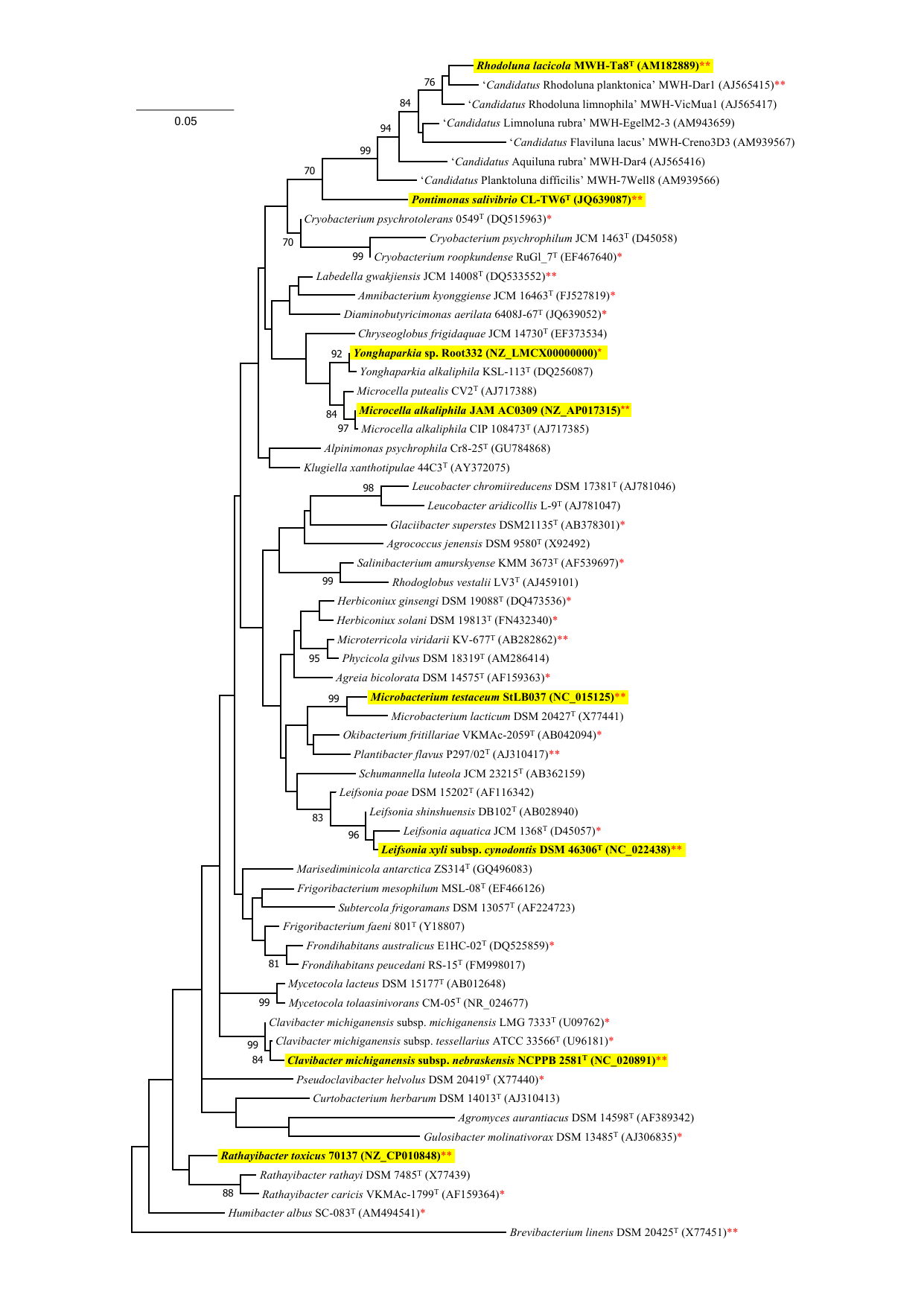
Figure S2. Maximum likelihood tree of 16S rRNA sequences placing representative genomes within the context of Microbacteriaceae type strains and other selected representatives. Two asterisks - complete genome available, One asterisk - incomplete genomic data available, Yellow highlight - representative genome used in this study. The tree was made with the MEGA package (Kumar et al., 2016). Only bootstrap values (expressed as percentage of 1000 replications) greater than 70% are shown at nodes. Bar, 0.05 nucleotide substitutions per site.

**Reference**

Kumar S, Stecher G, Tamura K. MEGA7: molecular evolutionary genetics analysis version 7.0 for bigger datasets. Mol Biol Evol. 2016; 33(7):1870–74.
