# Supplementary material for: Complete genome of streamlined marine actinobacterium Pontimonas salivibrio strain CL-TW6T adapted to coastal planktonic lifestyle
Source: BMC Genomics. 2018 Aug 22;19:625. doi: 10.1186/s12864-018-5019-9 (PMC6106888; doi:10.1186/s12864-018-5019-9)
Supplement: Supplementary file 5 — Table S3. A pair-wise comparison of selected genomes for (a) in silico DNA-DNA hybridization (DDH), (b) average amino acid identity (AAI), (c) δ* differences (the average difference of dinucleotide relative abundance), and (d) the percentage of conserved proteins (POCP). 1, Pontimonas salivibrio CL-TW6T; 2, Rhodoluna lacicola MWH-Ta8T; 3, Yonghaparkia sp. Root332; 4, Microcella alkaliphila JAM AC0309; 5, acMicro-4. (PDF 60 kb) [file 12864_2018_5019_MOESM5_ESM.pdf]

Table S3. A pair-wise comparison of selected genomes for (a) in silico DNA-DNA hybridization (DDH), (b) average amino acid identity (AAI), (c) δ^* differences (the average difference of dinucleotide relative abundance), and (d) the percentage of conserved proteins (POCP). 1, *Pontimonas salivibrio* CL-TW6^T; 2, *Rhodoluna lacicola* MWH-Ta8^T; 3, *Yonghaparkia* sp. Root332; 4, *Microcella alkaliphila* JAM AC0309; 5, acMicro-4.

(a) In silico DDH (%)[*]

	1	2	3	4	5
1 Pontimonas		**25.3**	**22.9**	**19.7**	**16.5**
2 Rhodoluna			**35.2**	**27.2**	**19.5**
3 Yonghaparkia				**20.6**	**19.2**
4 Microcella					**19.1**
5 acMicro-4					

(b) AAI (%)[**]

	1	2	3	4	5
1 Pontimonas		**52.2**	**57.2**	**57.2**	**63.8**
2 Rhodoluna			**55.5**	**55.1**	**54.3**
3 Yonghaparkia				**71.4**	**61.2**
4 Microcella					**60.8**
5 acMicro-4					

(c) δ^* differences (multiplied by 1000)[†]

	1	2	3	4	5
1 Pontimonas	**26**	**89**	**266**	**178**	**65**
2 Rhodoluna		**34**	**304**	**230**	**98**
3 Yonghaparkia			**30**	**101**	**225**
4 Microcella				**27**	**162**
5 acMicro-4					**20**

(d) POCP (%)[‡]

	1	2	3	4	5
1 Pontimonas		**52.1**	**51.5**	**51.7**	**67.0**
2 Rhodoluna			**47.9**	**46.0**	**56.3**
3 Yonghaparkia				**70.6**	**50.1**
4 Microcella					**48.0**
5 acMicro-4					

[*] Genome-to-genome distance calculator using BLAST+ [R1] (http://ggdc.dsmz.de/ggdc.php#)

[**] AAI calculator using reciprocal best hits [R2] (http://enve-omics.ce.gatech.edu/aai/)

[†] Campbell et al. [R3] (http://www.cmbl.uga.edu/software/delta-differences.html)

[‡] Qin et al. [R4]

References

R1. Meier-Kolthoff JP, Auch AF, Klenk HP, Göker M. Genome sequence-based species delimitation with confidence intervals and improved distance functions. BMC Bioinformatics. 2013;14:60.

R2. Rodriguez-R LM, Konstantinidis KT. Bypassing cultivation to identify bacterial species. Microbe. 2014;9(4):111–8.

R3. Campbell A, Mrázek J, Karlin S. Genome signature comparisons among prokaryote, plasmid, and mitochondrial DNA. Proc. Natl. Acad. Sci. USA. 1999:9184–9.

R4. Qin QL, Xie BB, Zhang XY, Chen XL, Zhou BC, Zhou J, Oren A, Zhang YZ. A proposed genus boundary for the prokaryotes based on genomic insight. J Bacterol. 2014;196(12):2210–5.
