# Supplementary material for: Complete genome of streamlined marine actinobacterium Pontimonas salivibrio strain CL-TW6T adapted to coastal planktonic lifestyle
Source: BMC Genomics. 2018 Aug 22;19:625. doi: 10.1186/s12864-018-5019-9 (PMC6106888; doi:10.1186/s12864-018-5019-9)
Supplement: Supplementary file 6 — Table S4. Distribution of best BlastP matches, %GC, genome size, and numbers of tRNA and rRNA genes in representative Actinobacteria genomes. (DOC 40 kb) [file 12864_2018_5019_MOESM6_ESM.doc]

Table S4. Distribution of best BlastP matches, %GC, genome size, and numbers of tRNA and rRNA genes in representative Actinobacteria genomes.

| Genus/assembly | Number of encoded proteins matching this entity in the top 3 BlastP matches keyed by *P. salivibrio* proteins^a^ | %GC | Genome size  (Mb) | #ORF | #tRNA | #rRNA |
| --- | --- | --- | --- | --- | --- | --- |
| CLTW6r |  | 58 | 1.8 | 1699 | 46 | 3 |
| acMicro-4 | 863 | 60 | 1.2 | 1987 |  |  |
| *Microcella* | 688 | 68 | 2.7 | 2652 | 45 | 3 |
| *Yonghaparkia* | 620 | 72 | >1.6 | 1529 | 27 | 3 |
| *Clavibacter* | 235 | 73 | 3.1 | 2819 | 47 | 6 |
| *Leifsonia* | 198 | 68 | 2.7 | 2215 | 47 | 3 |
| *Microbacterium* | 98 | 70 | 4.0 | 3585 | 45 | 6 |
| *Rathayibacter* | 94 | 61 | 2.3 | 2039 | 45 | 6 |
| *Rhodoluna* | 93 | 52 | 1.4 | 1364 | 39 | 3 |
| *Mycobacterium* | 53 | 66 | 4.4 | 3904 | 45 | 3 |
| *Streptomyces* | 47 | 73 | 6.3 | 5396 | 64 | 18 |
| MedAcidi -G1 | 2 | 42 | 1.9 | 1403 | 31 | 3 |

^a^The library consisted of proteins from all completely sequenced bacterial genomes, plus partially sequenced *Yonghaparkia*, and the full acMicro-4 and MedAcidi-G1 metagenomic assemblies.
