# Supplementary material for: Complete genome of streamlined marine actinobacterium Pontimonas salivibrio strain CL-TW6T adapted to coastal planktonic lifestyle
Source: BMC Genomics. 2018 Aug 22;19:625. doi: 10.1186/s12864-018-5019-9 (PMC6106888; doi:10.1186/s12864-018-5019-9)
Supplement: Supplementary file 7 — Figure S3. Phylogenomic tree. Encoded protein sequences catenated were selected from among large genes, with congruent single gene trees, and enriched with genes having BlastP results suggesting greater similarity of P. salivibrio with Yonghaparkia than to Microbacteria, Clavibacter, Rathayibacter, and Leifsonia. The computation is specifically designed to accept or reject a closer common ancestor with Yonghaparkia. Genes used were C3B54_11793, C3B54_11942, C3B54_11990, C3B54_111069, C3B54_111092, C3B54_111098, C3B54_111113, C3B54_111132, C3B54_111675. Time scale was determined as described in Fig. 2. (DOC 78 kb) [file 12864_2018_5019_MOESM7_ESM.doc]

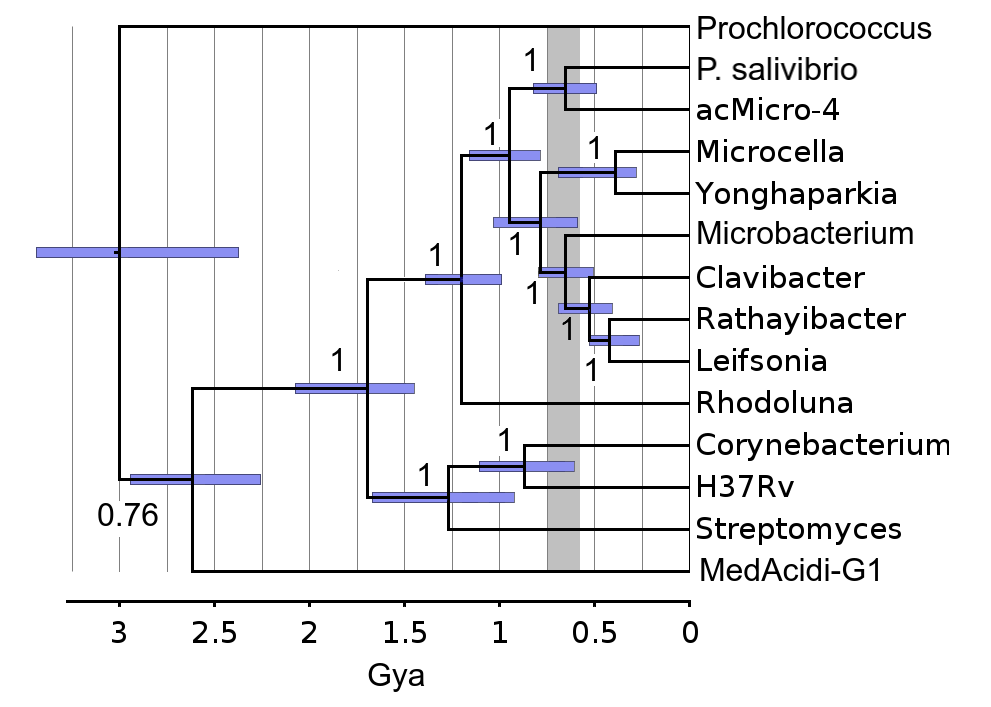


Figure S3. Phylogenomic tree. Encoded protein sequences catenated were selected from among large genes, with congruent single gene trees, and enriched with genes having BlastP results suggesting greater similarity of *P. salivibrio* with *Yonghaparkia* than to *Microbacteria*, *Clavibacter,* *Rathayibacter,* and *Leifsonia.* The computation is specifically designed to accept or reject a closer common ancestor with *Yonghaparkia*. Genes used were C3B54_11793, C3B54_11942, C3B54_11990, C3B54_111069, C3B54_111092, C3B54_111098, C3B54_111113, C3B54_111132, C3B54_111675. Time scale was determined as described in Fig. 2.
