# Supplementary material for: Complete genome of streamlined marine actinobacterium Pontimonas salivibrio strain CL-TW6T adapted to coastal planktonic lifestyle
Source: BMC Genomics. 2018 Aug 22;19:625. doi: 10.1186/s12864-018-5019-9 (PMC6106888; doi:10.1186/s12864-018-5019-9)
Supplement: Supplementary file 9 — Figure S4. Distribution of genes likely derived from horizontal transfer as indicated by low vertical index. A custom built BlastP library containing one entry for each gene in completely sequenced bacterial genomes was searched with each annotated P. salivibrio translated gene sequence, and the number of top 10 hits mapping to a genome classified as belonging to Microbacteriaceae was plotted in genomic order. (DOC 67 kb) [file 12864_2018_5019_MOESM9_ESM.doc]

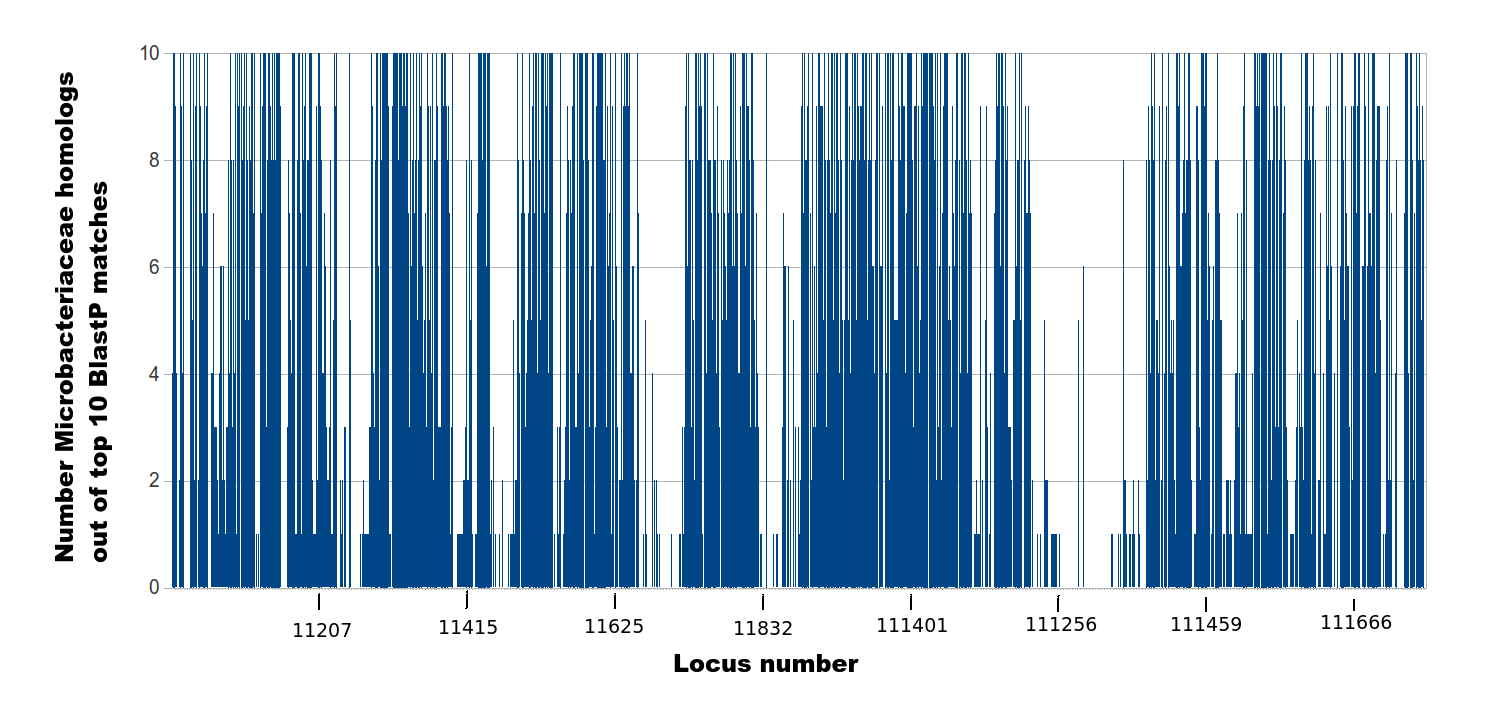


Figure S4. Distribution of genes likely derived from horizontal transfer as indicated by low vertical index. A custom built BlastP library containing one entry for each gene in completely sequenced bacterial genomes was searched with each annotated *P. salivibrio* translated gene sequence, and the number of top 10 hits mapping to a genome classified as belonging to Microbacteriaceae was plotted in genomic order.
