# Supplementary material for: Complete genome of streamlined marine actinobacterium Pontimonas salivibrio strain CL-TW6T adapted to coastal planktonic lifestyle
Source: BMC Genomics. 2018 Aug 22;19:625. doi: 10.1186/s12864-018-5019-9 (PMC6106888; doi:10.1186/s12864-018-5019-9)
Supplement: Supplementary file 11 — Table S7. Insertion sequences in P. salivibrio CL-TW6T. (DOC 40 kb) [file 12864_2018_5019_MOESM11_ESM.doc]

**Table S7. Insertion sequences in *P. salivibrio* CL-TW6^T^.**

| Element | Class^a^ | Coordinates | ORFs | IR | Target site dupl. | Organization |
| --- | --- | --- | --- | --- | --- | --- |
| ISPsa1 | IS3 | c(1711553 ..  1712823) | C3B54_111736,  C3B54_111735 | 26 bp GGG... | AGG | OrfAB^b^  Interrupts C3B54_111737 |
| ISPsa2 | IS3 | c(1257963  ..1259233) | C3B54_111293,  C3B54_111291 | 26bp  GGG... | - | OrfAB  no obvious interrupted gene |
| ISPsa6 | IS3 | Join(1263655 ..1263960,  1265208 ..  1266172) | C3B54_111302/C3B54_111302a,  C3B54_111305 | 26 bp  GGG... | - | OrfAB, interrupted by ISPsa5 |
| ISPsa3 | IS3 | c(1759568..  1760810) | C3B54_111784,  C3B54_111783 | 44 /43bp^c^  TGA... | GAGC | OrfAB  no obvious interrupted gene |
| ISPsa4 | IS3 | 1722156 ..1723398 | C3B54_111744,  C3B54_111745 | 44/43 bp  TGA... | CCTG | OrfAB  no obvious interrupted gene |
| ISPsa5 | IS3 | c(1263961 ..1265203) | C3B54_111304,  C3B54_111303 | 44/43 bp  TGA... | CAAC | OrfAB  Inserted in ISPsa6 |
| ISPsa7 | IS3?(defect-ive) | 829749 ..830963 | C3B54_11862,  C3B54_11681  (pseudogene) | Unrecog-nizable | - | OrfAB,  Defects in transposase frame, and no valid start codon or potential for programmed frameshift |
| ISPsa8 | IS481 | c(423002 .. 424242) | C3B54_11460 | 25 bp  AC... | - | Single ORF |
| ISPsa9 | IS481 | c(1717880  ..1719070) | C3B54_111741 | Unrecog-nizable | - | Single ORF |

^a^According to ISFinder (Siquier et al., 2012).

^b^OrfAB indicates an organization in which a programmed -1 frameshift within the first ORF (TnpA, mainly a DNA binding domain) would result in translation of the second ORF (TnpB, an integase homolog).

^c^For ISPSA3,4 and 5 there is a gap in the dowstream IR causing it to be one bp shorter than the upstream IR.

**Reference**

Siquier P, Varani A, Perochon J, Chandler M. Exploring bacterial insertion sequences with ISfinder: objectives, uses, and future developments. Methods Mol Biol. 2012;859:91–103.
